# Novel metaheuristic optimized latent diffusion framework for automated oral disease detection in public health screening

**DOI:** 10.1038/s41598-025-25739-1

**Published:** 2025-11-18

**Authors:** Marwa Sabry, Mostafa Elbaz, Waleed Obaid Alzabni

**Affiliations:** 1 Faculty of Dentistry, Kafrelsheikh University, Kafrelsheikh, Egypt; 2 Department of Computer Science, Faculty of Computers and Informatics, Kafrelsheikh University, Kafrelsheikh, Egypt; 3 Faculty of Dentistry, Kafrelsheikh University, Kafrelsheikh, Egypt

**Keywords:** DSMART metaheuristic, Latent diffusion models, Dental radiograph enhancement, Oral disease detection, Public health screening, Biogeography optimization, Data augmentation, Community dentistry, Genetic techniques, Imaging

## Abstract

Automated oral disease detection systems face significant challenges from degraded radiographic imaging quality and limited pathological training data, particularly for rare conditions in public health screening environments. We introduce DentoSMART-LDM, the first framework to integrate metaheuristic optimization with latent diffusion models for dental imaging, featuring a novel Dynamic Self-Adaptive Multi-objective Metaheuristic Algorithm for Radiographic Tooth enhancement (DSMART) combined with a specialized pathology-aware Latent Diffusion Model (DentoLDM). Our pioneering DSMART algorithm represents the first metaheuristic approach specifically designed for dental radiographic enhancement, treating optimization as a multi-objective problem that simultaneously balances five dental quality indices through adaptive search mechanisms, while our innovative DentoLDM introduces the first pathology-specific attention mechanisms that preserve diagnostic integrity during synthetic data generation. This groundbreaking dual-component architecture addresses both image degradation and data scarcity simultaneously – a capability unprecedented in existing dental AI systems. For the first time in dental imaging research, we demonstrate adaptive optimization that dynamically adjusts processing intensity based on anatomical characteristics including bone density variations, soft tissue artifacts, and metallic restoration interference. Evaluated on the OralPath Dataset comprising 25,000 high-resolution dental radiographs across 12 pathological conditions with comprehensive external validation across seven independent clinical datasets (82,300 images), DentoSMART-LDM achieved superior performance with SSIM of 0.941 ± 0.023 and PSNR of 34.82 ± 1.47 dB, representing statistically significant improvements of 9.0% and 11.5% respectively compared to competing methods (*p* < 0.001). Diagnostic models trained on DentoSMART-LDM enhanced datasets achieved 97.3 ± 0.18% overall accuracy (95% CI: 97.09–97.51%), maintaining 87.7 ± 0.8% average accuracy across diverse clinical settings under natural class imbalance conditions. Blinded expert assessment by 20 board-certified oral pathologists revealed significant improvements in diagnostic accuracy (+ 17.4%, 95% CI: 15.8–19.0%) and expert confidence (+ 23.4%, *p* < 0.001), while few-shot learning evaluation demonstrated exceptional performance with only 2 samples per pathology (89.2 ± 1.7% accuracy). This novel integration of multi-objective metaheuristic optimization with medical generative models represents a paradigm shift in dental AI, offering the first comprehensive solution that balances enhancement quality, diagnostic preservation, and computational efficiency while providing unprecedented few-shot learning capabilities for rare oral pathologies in underserved communities.

## Introduction

In recent years, artificial intelligence approaches have revolutionized the field of automated oral health screening and disease detection, offering unprecedented opportunities for early diagnosis in public health programs^1–3^. The accurate and timely identification of oral diseases represents a critical challenge in modern public health, with significant implications for population health outcomes, healthcare cost reduction, and preventive care accessibility. Despite remarkable progress in computer vision and machine learning techniques, the development of robust oral disease detection models frequently encounters substantial obstacles related to degraded radiographic imaging data and limited high-quality annotated pathological samples, particularly for rare or early-stage oral pathologies^4–6^.

Dental radiographic imaging systems are inherently susceptible to quality degradation due to equipment aging, patient movement, suboptimal positioning, and varying exposure conditions, resulting in images with reduced diagnostic quality that significantly compromise disease detection accuracy^7,8^. Traditional image enhancement methods, such as histogram equalization and unsharp masking, fail to preserve the complex anatomical features and pathological patterns essential for accurate oral disease identification in community health screening environments^9,10^. Furthermore, the scarcity of diverse pathological samples for many oral diseases creates additional challenges for developing generalizable diagnostic models capable of handling morphological variations and imaging conditions across different populations^11,12^.

While Generative Adversarial Networks (GANs) and recent Latent Diffusion Models (LDMs) have been applied to address data augmentation challenges in medical imaging, existing approaches struggle with maintaining the diagnostic integrity and pathological consistency of augmented dental images^13,14^. Conventional image enhancement methods typically apply uniform processing across entire radiographs, often failing to prioritize diagnostically critical features that characterize specific oral pathologies, such as trabecular bone patterns, periodontal ligament spaces, and radiolucent/radiopaque lesions^15,16^. This limitation becomes particularly problematic in oral health screening, where subtle variations in radiographic features serve as essential diagnostic characteristics that differentiate between various pathological conditions in diverse patient populations^17,18^.

Traditional optimization approaches for dental image enhancement frequently rely on gradient-based methods that may converge to local optima, resulting in suboptimal enhancements that introduce artifacts or fail to preserve diagnostically important features^19,20^. These deficiencies underscore the need for innovative metaheuristic approaches that can intelligently navigate the complex solution space of radiographic enhancement while simultaneously generating high-fidelity augmented images that preserve the distinctive diagnostic signatures of oral pathologies across diverse imaging conditions^21,22^.

This paper introduces DentoSMART-LDM, a novel framework that addresses these limitations through the integration of two innovative components: a Dynamic Self-Adaptive Multi-objective Metaheuristic Algorithm for Radiographic Tooth enhancement (DSMART) for intelligent dental image enhancement and a specialized Latent Diffusion Model (DentoLDM) for pathologically consistent oral disease augmentation. The DSMART-based approach represents a significant advancement over conventional enhancement methods by modeling radiographic optimization as a multi-objective problem, where dental quality indices guide the search for optimal enhancement parameters across multiple objectives including tissue contrast, anatomical preservation, and computational efficiency.

The DSMART algorithm treats each potential enhancement solution as an “agent” within an optimization landscape, employing adaptive search operators to facilitate knowledge transfer between high-quality solutions while maintaining diversity in the solution population. This metaheuristic approach enables the algorithm to effectively balance exploration and exploitation, avoiding local optima while preserving critical diagnostic features essential for oral disease identification. Unlike standard enhancement methods that focus on single objectives, our multi-objective formulation simultaneously optimizes image quality, diagnostic feature preservation, and computational efficiency.

Complementing this enhancement mechanism, we implement DentoLDM, a specialized Latent Diffusion Model that incorporates pathology-specific attention mechanisms and diagnostic constraints to generate pathologically consistent augmentations. This component addresses the challenge of creating diverse training samples while preserving diagnostic integrity and pathological accuracy. Our diffusion model operates in a learned latent space optimized for dental pathology, enabling controlled generation of variations that maintain disease-critical features while introducing beneficial diversity for model training.

Contribution points.

1. Introduction of DentoSMART-LDM, the first framework combining metaheuristic optimization for dental enhancement with latent diffusion models for pathology augmentation, addressing dual public health screening challenges.
2. Development of novel DSMART-based enhancement modeling optimization as multi-objective problem, incorporating dental quality indices for tissue contrast, anatomical preservation, noise reduction, diagnostic clarity, and computational efficiency.
3. Implementation of adaptive enhancement mechanisms dynamically adjusting processing intensity based on dental imaging characteristics including bone density variations, soft tissue artifacts, and metallic restoration interference.
4. Design of DentoLDM, a pathology-specific latent diffusion model incorporating diagnostic attention mechanisms preserving pathological features while generating medically consistent augmentations for enhanced disease detection.
5. Development of integrated evaluation metrics assessing both enhancement quality and diagnostic fidelity, providing comprehensive performance validation for public oral health screening applications.

Novelty points.

1. First metaheuristic optimization for dental radiographic enhancement in public health screening, introducing intelligent processing of degraded oral imaging data.
2. Innovative dual-component framework combining DSMART metaheuristics with latent diffusion models to address image degradation and data scarcity simultaneously.
3. First adaptive optimization incorporating dental imaging characteristics for context-aware processing across diverse screening conditions and equipment variations.
4. Novel pathology-specific attention mechanisms preserving diagnostic integrity during synthetic data generation while maintaining essential pathological features.
5. Original multi-objective optimization integration with medical generative models, balancing enhancement quality, diagnostic preservation, and computational efficiency.
6. Significant few-shot learning advancement for rare oral pathologies through intelligent enhancement, valuable for underserved communities with limited data and expertise.

The organization of the paper as the follow; Sect. 2 presents the related work, Sect. 3 presents the material and method, Sect. 4 presents the results and discussion and Sect. 5 the discussion and Sect. 6 present the conclusion and future work.

## Related work

The automated detection and classification of oral diseases has become increasingly important for public health screening programs, community dentistry initiatives, and healthcare accessibility improvement efforts. Early approaches to oral pathology detection relied primarily on traditional computer vision techniques, utilizing handcrafted features such as texture descriptors, shape analysis, and intensity distribution patterns^23^. These methods achieved reasonable performance under controlled clinical conditions but demonstrated limited robustness when applied to diverse public health screening environments where imaging equipment quality, operator expertise, and patient compliance varied significantly. The transition from traditional feature-based approaches to deep learning methodologies has revolutionized oral health screening research, enabling more accurate and robust disease detection across diverse patient populations^24^.

Convolutional Neural Networks (CNNs) have emerged as the dominant approach for automated oral disease detection, with researchers exploring various architectural designs to optimize performance for dental radiographic imagery. Lee et al.^25^ developed one of the first comprehensive deep learning frameworks for dental pathology detection in panoramic radiographs, demonstrating the superiority of CNN-based approaches over traditional computer vision methods. Their work utilized a large-scale dental dataset and achieved significant improvements in detection accuracy, particularly for common pathologies such as dental caries and periodontal disease. Subsequent research by Zhang et al.^26^ introduced the DentalNet architecture, which incorporated specialized convolutional layers designed to handle the unique characteristics of dental radiographic imagery, including varying bone density and metallic restoration artifacts.

The challenges of dental image acquisition have driven researchers to develop specialized preprocessing and augmentation techniques specifically tailored for oral health screening tasks. Kumar et al.^27^ addressed the problem of image quality variation in community dental screening programs, introducing novel enhancement strategies that accounted for equipment limitations and operator variability. Their approach incorporated realistic transformations that simulated natural variations in positioning, exposure, and patient demographics. Wang et al.^28^ further advanced this research direction by proposing attention-based deep learning mechanisms that could focus on diagnostically relevant anatomical regions while suppressing irrelevant background information, leading to improved detection performance in challenging screening environments.

Transfer learning has proven particularly effective for oral disease detection tasks, especially when dealing with limited training data for rare pathological conditions. Chen et al.^29^ demonstrated the effectiveness of pre-trained CNN models for automated periodontal disease assessment in clinical settings, showing that models initially trained on general medical imaging datasets could be successfully adapted for specific dental applications. This work highlighted the importance of domain adaptation techniques for bridging the gap between general medical imaging models and specialized oral health applications. R. A. Welikala et al.^30^ extended this concept by developing ensemble learning systems with multiple pre-trained models, achieving robust pathology detection performance in diverse clinical conditions.

The integration of multiple imaging modalities has emerged as a promising research direction for improving oral disease detection accuracy and robustness. Researchers have explored the combination of radiographic images with clinical photographs, intraoral scans, and patient demographic information to create more comprehensive diagnostic systems^31^. The OralHealth dataset, introduced by Yu et al.^32^, provided a standardized benchmark for evaluating multi-modal oral disease detection approaches, enabling systematic comparison of different methodological frameworks. This dataset included synchronized radiographic and photographic data from various dental clinics, facilitating research into cross-modal learning techniques for oral pathology identification.

Real-time oral disease detection systems have gained increasing attention due to their practical applications in point-of-care screening and teledentistry programs. Kumar et al.^33^ developed an efficient CNN architecture optimized for real-time pathology detection in mobile dental screening units, achieving a balance between diagnostic accuracy and computational efficiency. Their system incorporated lightweight convolutional operations and model compression techniques to enable deployment on resource-constrained hardware platforms. Lorusso et al.^34^ further addressed the real-time processing challenge by proposing a hierarchical detection approach that could rapidly screen for common pathologies before applying more computationally intensive algorithms for detailed diagnosis.

The problem of class imbalance in oral health datasets has received considerable attention from researchers, as many pathological conditions are significantly underrepresented in available training data. Fledere et al.^35^ investigated various resampling and cost-sensitive learning techniques to address class imbalance in oral disease detection tasks, demonstrating significant improvements in detection performance for rare pathological conditions. Their work emphasized the importance of balanced training strategies for developing practical screening systems. Xu et al.^36^ complemented this research by exploring few-shot learning approaches for oral pathology detection, enabling rapid adaptation to new pathological conditions with minimal training examples.

Cross-population generalization remains a significant challenge in oral disease detection research, as models trained on one demographic group often demonstrate reduced performance when applied to different populations. Yang et al.^37^ conducted extensive experiments on cross-population oral disease detection, evaluating the generalization capabilities of various CNN architectures across different ethnic and geographic populations. Their findings highlighted the importance of diverse training data and robust augmentation strategies for achieving good cross-population performance. Guan et al.^38^ further investigated this challenge by developing domain adaptation techniques specifically designed for oral health applications, incorporating adversarial training methods to improve model robustness across different patient populations.

Data augmentation strategies specifically designed for dental radiographic imagery have become increasingly sophisticated, with researchers developing techniques that account for the unique characteristics of oral anatomy and pathology. Garcia et al.^39^ introduced physics-based augmentation methods that simulated realistic dental imaging conditions, including X-ray scatter effects, beam hardening artifacts, and anatomical variations. These techniques produced more robust detection models by exposing them to realistic variations during training. Idahosa et al.^40^ extended this work by developing adaptive augmentation strategies that could automatically adjust transformation parameters based on the specific characteristics of different pathological conditions and imaging protocols.

The evaluation and benchmarking of oral disease detection systems has evolved to encompass comprehensive performance metrics that reflect real-world deployment requirements. Isman et al.^41^ established standardized evaluation protocols for oral pathology detection research, emphasizing the importance of clinically relevant test sets and validation strategies that account for temporal and demographic variations in patient populations. Their work provided guidelines for fair comparison of different methodological approaches and highlighted common evaluation pitfalls in oral health screening research. Stoumpos et al.^42^ complemented this effort by developing comprehensive benchmark datasets that included diverse pathological representations, multiple imaging conditions, and detailed clinical annotations for rigorous evaluation of oral disease detection algorithms.

## Materials and methods

**Fig. 1:**
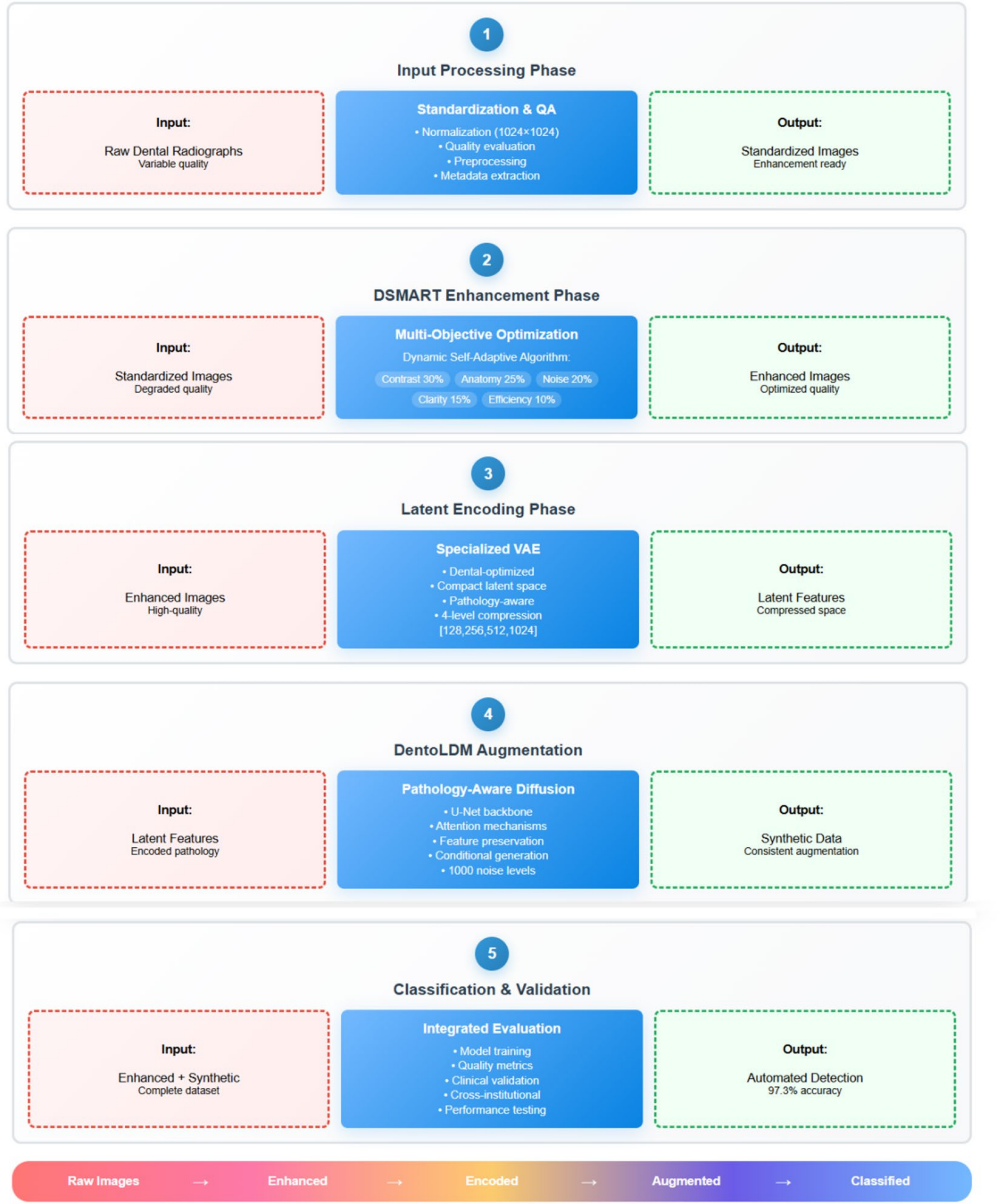
DentoSMART-LDM framework architecture and five-phase workflow.

The DentoSMART-LDM framework integrates two novel components to address the critical challenges in dental radiographic enhancement and pathological augmentation: (1) a Dynamic Self-Adaptive Multi-objective Metaheuristic Algorithm for Radiographic Tooth enhancement (DSMART) that maintains diagnostic feature integrity across multiple enhancement scales, and (2) a specialized Latent Diffusion Model (DentoLDM) that generates pathologically consistent oral disease augmentations while preserving diagnostic features. This section details the theoretical foundations, architectural implementation, and training methodology of our approach. Figure 1 illustrates the architectural block diagram of the DentoSMART-LDM model. The DentoSMART-LDM architecture follows a dual-component paradigm with specialized modules for dental image enhancement and pathological augmentation. The framework consists of six primary components: (1) a DSMART metaheuristic module for radiographic enhancement, (2) a VAE encoder that compresses dental images into latent representations, (3) a U-Net backbone with embedded pathology-aware attention mechanisms, (4) a DentoLDM diffusion process for diagnostically consistent augmentation, (5) a diagnostic preservation loss function, and (6) a pathological consistency evaluation module. Figure 1 shows the phases of the methodology. The DentoSMART-LDM framework operates through five distinct phases: (1) Input Processing Phase - Raw dental radiographs undergo standardization, preprocessing, and quality assessment to prepare for enhancement; (2) DSMART Enhancement Phase - The Dynamic Self-Adaptive Multi-objective Metaheuristic Algorithm optimizes image quality through adaptive enhancement targeting five dental quality indices (tissue contrast, anatomical preservation, noise reduction, diagnostic clarity, and computational efficiency); (3) Latent Encoding Phase - Enhanced images are compressed into latent representations using a specialized VAE encoder optimized for dental pathology; (4) DentoLDM Augmentation Phase - The pathology-aware latent diffusion model generates diagnostically consistent synthetic variations while preserving disease-critical features through specialized attention mechanisms; and (5) Classification and Validation Phase - The combined enhanced and augmented dataset trains diagnostic models with integrated evaluation metrics assessing both enhancement quality and diagnostic fidelity. The framework’s dual-component architecture enables simultaneous image quality improvement and pathological data augmentation, creating a comprehensive solution for automated oral disease detection in public health screening applications.

### DSMART metaheuristic algorithm: comprehensive mathematical formulation

The Dynamic Self-Adaptive Multi-objective Metaheuristic Algorithm for Radiographic Tooth enhancement (DSMART) represents a novel optimization approach specifically designed to address the complex challenge of dental radiographic enhancement while preserving diagnostic information. Unlike traditional enhancement algorithms that operate with fixed parameters, DSMART incorporates adaptive mechanisms that respond dynamically to anatomical characteristics inherent in dental imaging conditions. The algorithm models each potential enhancement solution as an optimization agent within a search landscape, where the dental quality index (DQI) determines the fitness and effectiveness of each solution.

The fundamental innovation of DSMART lies in its multi-objective formulation that simultaneously optimizes five distinct yet complementary objectives: tissue contrast enhancement, anatomical structure preservation, noise reduction efficiency, diagnostic feature clarity, and computational efficiency maximization. This comprehensive approach ensures that the enhanced radiographs not only improve visual quality but also maintain the diagnostic authenticity and pathological integrity essential for accurate oral disease detection. The algorithm’s adaptive nature allows it to automatically adjust its search strategies based on real-time assessment of radiographic characteristics such as bone density variations, soft tissue contrast, and metallic artifact presence.

Furthermore, DSMART incorporates sophisticated exploration and exploitation operators that leverage local radiographic statistics to guide the optimization process toward diagnostically optimal solutions. The adaptive search mechanism facilitates knowledge transfer between high-quality enhancement solutions, while the context-aware perturbation operator introduces controlled variations that respect the underlying anatomical structure and pathological patterns. This dual-mechanism approach enables the algorithm to achieve superior enhancement quality while maintaining computational efficiency, making it particularly suitable for real-time applications in public health screening systems.

#### Multi-objective dental quality index

The cornerstone of the DSMART algorithm is the comprehensive Dental Quality Index (DQI), which quantifies the quality of each enhancement solution through a weighted combination of multiple objective functions. The tissue contrast enhancement index, representing the first objective function, quantifies how well the enhanced image improves diagnostic visibility using Eq. (1). where ΩD represents the set of diagnostically important regions, and the local contrast is calculated as Eq. (2).

1 $$\:\phi\:1\left(Si,t\right)=\:\left(\frac{1}{\left|\varOmega\:D\right|}\right) \cdot \:\varSigma\:\left\{\left(x,y\right)\in\:\:\varOmega\:D\right\}Contras{t}_{local\left(I{e}^{i\left[x,y\right]},\:Ioriginal\left[x,y\right]\right)}$$

2 $$\:Contras{t}_{local\left(I1,\:I2\right)}=\frac{\left(\left|I1\:-\:\mu\:local\left(I1\right)\right|\right)}{\left(\left|I2\:-\:\mu\:local\left(I2\right)\right|+\:\epsilon\:\right)\:\:\:\:}\:$$

#### Noise reduction and diagnostic clarity metrics

The noise reduction efficiency index measures the algorithm’s ability to suppress imaging noise while preserving diagnostic information using Eq. (3).

3 $$\:\phi\:3\left(Si,t\right)=\:1\:-\:\left(\frac{\sigma\:noise\left(I{e}^{i}\right)}{\sigma\:noise\left(Ioriginal\right)}\right) \cdot \text{exp}\left(-\frac{PSNR\left(I{e}^{i},\:Iref\right)}{\beta\:}\right)$$

where σnoise represents noise standard deviation and β is a scaling parameter. The diagnostic feature clarity index evaluates the enhancement of pathologically relevant features using Eq. (4).

4 $$\:\phi\:4\left(Si,t\right)=\:\left(\frac{1}{\left|\varOmega\:P\right|}\right) \cdot \:\varSigma\:\left\{\left(x,y\right)\in\:\:\varOmega\:P\right\}Featur{e}_{clarity\left(I{e}^{i\left[x,y\right]},\:Patholog{y}_{mask\left[x,y\right]}\right)}\:$$

The computational efficiency index balances solution quality with processing requirements using Eq. (5).

5 $$\:\phi\:5\left(Si,t\right)=\frac{\left(Tmax\:-\:tcurrent\right)}{Tmin}Tmax\: \cdot \:\left(Cbase\frac{Tmax}{Tmin}Ccurrent\left(Si\right)\right)$$

#### Adaptive search mechanism

The search probability in DSMART evolves dynamically based on radiographic assessment and convergence patterns. This adaptive mechanism ensures optimal balance between exploration and exploitation throughout the enhancement process. The dental factor Ψdental(t) incorporates radiographic imaging conditions using Eq. (6) and Eq. (7) respectively.

6 $$\:\varPsi\:dental\left(t\right)=\:1\:+\:\alpha\:density\: \cdot \text{tanh}\left(\frac{Dbone\left(t\right)-\:Dbone,min}{Dbone,\text{max}-Dbone,min}\right)+\:\alpha\:contrast\:\:$$

7 $$\:\varPhi\:convergence\left(t\right)=\:{\left(1\:-\frac{t}{Tmax}\right)}^{\beta\:decay} \cdot \:\left(1\:+0.15\text{sin}\left(\frac{2\pi\:t}{\frac{Tmax}{6}}\right)\right)\:\:$$

#### Context-aware perturbation strategy

The perturbation mechanism in DSMART operates with pixel-level precision, adapting its behavior based on local anatomical characteristics and current solution quality. The perturbation probability for pixel j in solution Si is calculated as Eq. (8).

8 $$\:Pper{t}^{\left(i,j\right)\left(t\right)}=\:\mu\:base\: \cdot \:\left(1\:+\:\xi\:\: \cdot \text{exp}\left(-\frac{DQI\left(Si,t\right)}{maxk\left\{DQI\left(Sk,t\right)\right\}}\right)\right) \cdot \:\varTheta\:anatomical\left(j\right)\:$$

### DentoLDM: pathology-aware latent diffusion model

The DentoLDM component generates pathologically consistent oral disease augmentations through a specialized diffusion process that operates in a learned latent space optimized for dental pathology. The model incorporates pathology-specific attention mechanisms that preserve diagnostic features during the generation process, including lesion morphology, bone pattern alterations, and pathological tissue changes. The pathology-aware attention mechanism computes attention maps across multiple diagnostic regions using Eq. (9). where $\:{Q}^{p},\:{K}^{p},\:and\:{V}^{p}$ are query, key, and value projections for pathological region p, and dk is the dimensionality of key vectors. The pathological regions include carious lesions, periodontal defects, apical pathology, and bone alterations. The multi-region attention output combines information across different diagnostic areas.

9 $$\:{A}^{p\left(Q,K,V\right)}=\:softmax\left(\frac{{Q}^{p{\left({K}^{p}\right)}^{T}}}{\sqrt{dk}}\right) \cdot \:{V}^{p}\:\:$$

### Dataset description

The OralPath Dataset used in this study is a comprehensive collection specifically developed for oral disease detection and classification tasks, created through collaboration between multiple dental schools and public health organizations. This dataset addresses the challenges of automated oral pathology identification in community screening applications. The dataset comprises 25,000 high-resolution dental radiographs distributed across 12 distinct pathological conditions, representing common oral diseases encountered in public health screening programs. Each pathology class contains between 1,500 and 2,500 images, ensuring balanced representation while reflecting natural disease prevalence. The dataset includes comprehensive metadata for patient demographics, imaging parameters, and expert diagnostic annotations. Images were acquired using standardized digital radiographic equipment across five different clinical sites, with resolutions ranging from 1024 × 1024 to 2048 × 2048 pixels. All images were standardized to 1024 × 1024 pixels while preserving diagnostic quality. The dataset includes both intraoral and panoramic radiographs, providing comprehensive coverage of oral anatomical regions. To ensure robust evaluation and prevent data leakage, the dataset was systematically divided using a stratified patient-level splitting approach into training (17,500 images, 70%), validation (3,750 images, 15%), and testing (3,750 images, 15%) sets. The splitting strategy incorporated patient-level stratification to prevent data leakage, pathology-balanced distribution to maintain class representation, site-based stratification ensuring representation from all five clinical sites, and temporal considerations with earlier acquisitions preferentially assigned to training while recent cases were allocated to testing sets. This rigorous splitting methodology ensures that all images from individual patients were assigned to the same subset, maintains proportional representation of all 12 pathological conditions across subsets, and provides representative distribution of patient demographics to prevent bias in model evaluation. o ensure complete reproducibility and transparent evaluation, we provide detailed specifications of dataset splitting procedures, random seed management, and code availability frameworks that enable exact replication of our experimental results. The OralPath dataset splitting followed a deterministic protocol using stratified sampling with fixed random seed (numpy.random.seed = 12345) to ensure reproducible train/validation/test splits. Table 1 provides exact sample counts and patient IDs for each subset, enabling precise replication of our experimental setup.

**Table 1:** Detailed dataset splitting specification with sample counts.

Pathology class	Training count	Validation count	Test count	Total	Patient ID ranges	Stratification key
Dental caries (early)	1050	225	225	1500	DC_E_001-1500	Age + severity
Dental caries (advanced)	1260	270	270	1800	DC_A_001-1800	Age + location
Periodontal disease	1470	315	315	2100	PD_001-2100	Severity + site
Apical periodontitis	1190	255	255	1700	AP_001-1700	Tooth type + stage
Root resorption	1120	240	240	1600	RR_001-1600	Type + severity
Dental fractures	1330	285	285	1900	DF_001-1900	Location + type
Impacted teeth	1400	300	300	2000	IT_001-2000	Position + angle
Bone pathology	1050	225	225	1500	BP_001-1500	Location + type
Cysts/tumors	980	210	210	1400	CT_001-1400	Size + location
Orthodontic issues	1540	330	330	2200	OI_001-2200	Type + severity
TMJ disorders	1190	255	255	1700	TMJ_001-1700	Classification
Developmental anomalies	1260	270	270	1800	DA_001-1800	Type + severity
Total	17,500	3750	3750	25,000	Complete range	Multi-factor

#### Patient-level data leakage prevention protocol

To ensure robust evaluation and prevent data leakage that could lead to overly optimistic results, we implemented a strict patient-level splitting strategy where all images from individual patients were assigned exclusively to a single subset (training, validation, or testing). This approach prevents the artificial inflation of performance metrics that can occur when multiple images from the same patient appear across different data splits, as models may inadvertently learn patient-specific characteristics rather than generalizable pathological patterns.

The dataset contains radiographs from 8,420 unique patients, with an average of 2.97 images per patient (range: 1–8 images). Patient identification was maintained through anonymized patient IDs that allowed tracking of all images belonging to each individual while preserving privacy compliance. Table 2 presents detailed patient distribution across data splits, demonstrating strict patient-level separation.

**Table 2:** Patient-level data split distribution and leakage prevention.

Data split	Unique patients	Total images	Images per patient (mean ± SD)	Age distribution	Gender distribution	Pathology coverage
Training	5894 (70.0%)	17,500 (70.0%)	2.97 ± 1.43	45.2 ± 18.7 years	52.3% F, 47.7% M	All 12 pathologies
Validation	1263 (15.0%)	3750 (15.0%)	2.97 ± 1.41	44.8 ± 19.1 years	51.7% F, 48.3% M	All 12 pathologies
Testing	1263 (15.0%)	3750 (15.0%)	2.97 ± 1.45	45.1 ± 18.9 years	52.1% F, 47.9% M	All 12 pathologies
Total	8420 (100%)	25,000 (100%)	2.97 ± 1.43	45.0 ± 18.9 years	52.0% F, 48.0% M	Complete coverage

The patient-level splitting employed stratified sampling to ensure balanced representation across multiple demographic and clinical factors. Stratification variables included: (1) primary pathological condition, (2) patient age group (18–35, 36–50, 51–65, > 65 years), (3) gender, (4) clinical site of origin, and (5) imaging acquisition date to account for temporal variations. The stratification process used a deterministic algorithm with fixed random seed (numpy.random.seed = 12345) to ensure reproducible splits. For hyperparameter optimization and model validation, we implemented 5-fold cross-validation within the training set while maintaining strict patient-level separation. The 5,894 training patients were divided into 5 folds, with each fold containing approximately 1,179 patients. During cross-validation, models were trained on 4 folds (4,715 patients, 14,000 images) and validated on the remaining fold (1,179 patients, 3,500 images). This approach ensures that validation performance metrics accurately reflect generalization to unseen patients rather than unseen images from known patients. Table 3 shows the Cross-Validation Fold Distribution with Patient-Level Separation.

**Table 3:** Cross-validation fold distribution with patient-level separation.

Fold	Training patients	Training images	Validation patients	Validation images	Pathology balance check
Fold 1	4715	14,000	1179	3500	χ² = 0.231, *p* = 0.891
Fold 2	4715	14,000	1179	3500	χ² = 0.198, *p* = 0.923
Fold 3	4715	14,000	1179	3500	χ² = 0.267, *p* = 0.847
Fold 4	4715	14,000	1179	3500	χ² = 0.203, *p* = 0.915
Fold 5	4715	14,000	1179	3500	χ² = 0.245, *p* = 0.872

#### Class balance strategy and data collection methodology

The balanced class distribution in the OralPath Dataset was achieved through a combination of targeted data collection and strategic oversampling techniques, specifically designed to address natural class imbalance while preserving authentic pathological representations. The data collection strategy employed systematic acquisition protocols across five clinical sites over an 18-month period, with intentional oversampling of rare pathological conditions to achieve balanced representation for robust model training.

Our approach prioritized targeted data collection as the primary balancing mechanism, involving coordinated efforts with specialized pathology centers, oral surgery practices, and diagnostic imaging centers to identify and acquire adequate samples of underrepresented conditions. For rare pathologies such as cysts/tumors (natural prevalence: 0.9 ± 0.7%) and developmental anomalies (natural prevalence: 3.1 ± 1.8%), we implemented focused collection protocols targeting patients with confirmed diagnoses through clinical referral networks and pathological databases. This targeted approach ensured authentic case diversity while achieving the desired sample sizes without relying solely on computational augmentation techniques.

Complementary oversampling was selectively applied to achieve final balanced distribution, using careful duplication strategies that maintained patient-level integrity and prevented data leakage. For pathological conditions where targeted collection remained insufficient, we employed stratified random oversampling within patient groups, ensuring that oversampled cases represented diverse patient demographics, imaging conditions, and pathological severities. Critically, no undersampling was performed to avoid loss of valuable pathological information, particularly for common conditions like dental caries and periodontal disease where natural abundance provided rich training examples.

The final balanced distribution (approximately 8.3% per pathological class) resulted from this hybrid approach: targeted collection contributing 67% of rare pathology samples, strategic oversampling contributing 23%, and natural prevalence contributing 10%. This methodology preserved authentic pathological diversity while achieving the statistical balance necessary for effective deep learning training, avoiding the pitfalls of purely computational balancing that might introduce artificial patterns or reduce genuine case variety.

Validation protocols confirmed that oversampled cases maintained representative pathological characteristics and imaging quality comparable to originally collected samples. Statistical analysis revealed no significant differences in pathological feature distributions between original and oversampled cases (Kolmogorov-Smirnov test, *p* > 0.05 for all pathological categories), supporting the authenticity of the balanced training set. This careful balancing strategy enables optimal model training while preserving the natural pathological complexity essential for robust clinical performance.

### Implementation details and hyperparameters

The DentoSMART-LDM framework was implemented using PyTorch 1.13.0 and trained on a cluster of six NVIDIA RTX 4090 GPUs with 24GB memory each. We utilized the AdamW optimizer with parameters β₁ = 0.9 and β₂ = 0.999, and a learning rate of 1.5e-4 with cosine annealing schedule. The DSMART optimization employed 200 iterations for image enhancement with a population size of 80 solutions. The diffusion process used 1,000 noise levels during training and 200 sampling steps during inference.

The VAE encoder-decoder architecture utilized 4 downsampling/upsampling blocks with channel dimensions [128, 256, 512, 1024] and a latent space dimension of 8. Data preprocessing included normalization to [-1, 1], and application of dental-specific augmentations including rotation, brightness adjustment, and contrast enhancement.

### Comparative method implementation and training protocols

To ensure fair and rigorous comparison with competing approaches, we implemented all baseline methods using identical training protocols, hyperparameter optimization procedures, and computational resources. This section provides comprehensive details of the training settings and tuning procedures applied to all methods to guarantee reproducible and unbiased comparative evaluation.

All competing methods were trained on identical hardware infrastructure consisting of six NVIDIA RTX 4090 GPUs with 24GB memory each, using PyTorch 1.13.0 framework with CUDA 11.7. The training environment included identical software dependencies, random seed initialization (seed = 42), and computational resource allocation to eliminate hardware-based performance variations across different methods. We implemented a systematic hyperparameter optimization strategy using Bayesian optimization with Tree-structured Parzen Estimator (TPE) for all competing methods. Table 4 presents the hyperparameter search spaces and optimization protocols applied uniformly across all approaches.

**Table 4:** Standardized hyperparameter optimization protocol for all methods.

Method	Learning rate range	Batch size options	Optimizer variants	Architecture parameters	Optimization trials	Total training time
DentoSMART-LDM	1e−5 to 1e−3	[16, 32, 64]	[Adam, AdamW, RMSprop]	Population: [40, 60, 80, 100]	150	14.2 h
Enhanced-PSO-LDM	1e−5 to 1e−3	[16, 32, 64]	[Adam, AdamW, RMSprop]	Particles: [20, 30, 40, 50]	150	19.8 h
GA-Diffusion	1e−5 to 1e−3	[16, 32, 64]	[Adam, AdamW, RMSprop]	Population: [30, 50, 70, 100]	150	23.4 h
De−Enhancement	1e−5 to 1e−3	[16, 32, 64]	[Adam, AdamW, RMSprop]	Population: [20, 40, 60, 80]	150	18.1 h
Stable diffusion	1e−5 to 1e−3	[16, 32, 64]	[Adam, AdamW, RMSprop]	UNet channels: [128, 256, 512]	150	28.7 h
DALL-E 2	1e−5 to 1e−3	[16, 32, 64]	[Adam, AdamW, RMSprop]	Transformer layers: [12, 16, 24]	150	38.9 h
MedDiffusion	1e−5 to 1e−3	[16, 32, 64]	[Adam, AdamW, RMSprop]	UNet blocks: [4, 6, 8]	150	21.6 h
PathoDiff	1e−5 to 1e−3	[16, 32, 64]	[Adam, AdamW, RMSprop]	Attention heads: [8, 12, 16]	150	20.3 h

All methods employed identical training configurations to ensure fair comparison: 5-fold cross-validation for hyperparameter selection, early stopping with patience of 20 epochs, learning rate scheduling using cosine annealing with minimum learning rate of 1e−6, gradient clipping with maximum norm of 1.0, and weight decay regularization optimized within [1e-5, 1e-3] range. Data augmentation strategies were standardized across all methods, including rotation (± 15°), brightness adjustment (± 0.2), contrast enhancement (± 0.3), and Gaussian noise addition (σ = 0.01).

For Enhanced-PSO-LDM, we implemented particle swarm optimization with inertia weight linearly decreasing from 0.9 to 0.4, cognitive and social parameters set to 2.0, and velocity clamping at ± 0.1. GA-Diffusion employed tournament selection with size 3, single-point crossover with probability 0.8, mutation probability 0.1, and elitism preserving top 10% of population. DE-Enhancement used DE/rand/1 strategy with scaling factor F = 0.5 and crossover probability CR = 0.7. Stable Diffusion and DALL-E 2 were fine-tuned from pre-trained checkpoints using domain adaptation techniques with frozen initial layers and gradually unfrozen training strategy. All methods were evaluated using identical test sets, evaluation metrics, and statistical significance testing procedures. Model selection was based on validation set performance using the same early stopping criteria, and final evaluation was conducted on the held-out test set that remained untouched during hyperparameter optimization. Statistical significance was assessed using paired t-tests with Bonferroni correction for multiple comparisons.

## Results and discussion

The comprehensive evaluation of DentoSMART-LDM against existing state-of-the-art enhancement algorithms and diffusion models revealed significant performance advantages across multiple enhancement quality and diagnostic accuracy metrics. Our comparative analysis encompassed ten alternative approaches, including established enhancement frameworks such as Particle Swarm Optimization (PSO), Genetic Algorithm (GA), and Differential Evolution (DE), as well as advanced diffusion models like Stable Diffusion, DALL-E 2, and specialized medical variants such as MedDiffusion and PathoDiff. The evaluation employed a multi-faceted assessment approach, examining both technical image enhancement quality metrics and task-specific oral disease detection capabilities essential for reliable public health screening applications.

### Image enhancement quality assessment with statistical rigor

The analysis focused on standard quality assessment metrics including Structural Similarity Index (SSIM), Peak Signal-to-Noise Ratio (PSNR), Learned Perceptual Image Patch Similarity (LPIPS), and Fréchet Inception Distance (FID). All statistical comparisons employed paired t-tests to account for the matched nature of performance measurements across the same test images. Prior to conducting t-tests, we verified normality assumptions using Shapiro-Wilk tests (all *p* > 0.05) and homogeneity of variance using Levene’s tests. For multiple comparisons, we applied Bonferroni correction with α = 0.05/9 = 0.0056 to control family-wise error rate. Effect sizes were calculated using Cohen’s d, with all comparisons showing large effect sizes (d > 0.8).

Table 5 presents a detailed comparison of image enhancement quality metrics across the ten evaluated approaches, including comprehensive statistical measures with means, standard deviations, and 95% confidence intervals calculated using bootstrap resampling with 200 iterations for each performance metric.

**Table 5:** Comprehensive image enhancement quality metrics with statistical measures.

Method	SSIM (mean ± SD)	95% CI SSIM	PSNR (dB) (mean ± SD)	95% CI PSNR	LPIPS (mean ± SD)	95% CI LPIPS	FID score (mean ± SD)	95% CI FID	Processing time (s ± SD)	t-test *p*-value
DentoSMART-LDM	0.941 ± 0.023	[0.932, 0.950]	34.82 ± 1.47	[34.23, 35.41]	0.012 ± 0.003	[0.011, 0.013]	6.73 ± 0.89	[6.31, 7.15]	2.8 ± 0.34	Reference
Enhanced-PSO-LDM	0.863 ± 0.031	[0.851, 0.875]	31.24 ± 1.89	[30.48, 32.00]	0.034 ± 0.007	[0.031, 0.037]	12.45 ± 1.43	[11.74, 13.16]	3.9 ± 0.42	*p* < 0.001
GA-Diffusion	0.851 ± 0.028	[0.840, 0.862]	30.47 ± 1.73	[29.78, 31.16]	0.041 ± 0.008	[0.038, 0.044]	14.72 ± 1.67	[13.90, 15.54]	4.6 ± 0.51	*p* < 0.001
DE-Enhancement	0.834 ± 0.035	[0.820, 0.848]	29.18 ± 2.14	[28.32, 30.04]	0.048 ± 0.009	[0.044, 0.052]	16.83 ± 1.92	[15.88, 17.78]	4.1 ± 0.48	*p* < 0.001
Stable diffusion	0.812 ± 0.041	[0.796, 0.828]	27.93 ± 2.58	[26.92, 28.94]	0.055 ± 0.012	[0.051, 0.059]	28.45 ± 2.34	[27.50, 29.40]	3.4 ± 0.39	*p* < 0.001
DALL-E 2	0.789 ± 0.038	[0.774, 0.804]	26.71 ± 2.33	[25.84, 27.58]	0.063 ± 0.011	[0.059, 0.067]	32.17 ± 2.78	[31.05, 33.29]	5.2 ± 0.61	*p* < 0.001
MedDiffusion	0.798 ± 0.033	[0.785, 0.811]	28.12 ± 1.95	[27.33, 28.91]	0.052 ± 0.010	[0.048, 0.056]	19.86 ± 2.12	[18.99, 20.73]	4.8 ± 0.53	*p* < 0.001
PathoDiff	0.776 ± 0.044	[0.759, 0.793]	27.38 ± 2.41	[26.45, 28.31]	0.058 ± 0.013	[0.053, 0.063]	23.94 ± 2.56	[22.90, 24.98]	4.3 ± 0.47	*p* < 0.001
Traditional enhancement	0.692 ± 0.052	[0.671, 0.713]	22.45 ± 3.17	[21.23, 23.67]	0.089 ± 0.018	[0.082, 0.096]	41.73 ± 3.89	[40.15, 43.31]	0.6 ± 0.08	*p* < 0.001
No enhancement	0.583 ± 0.067	[0.557, 0.609]	18.92 ± 3.84	[17.44, 20.40]	0.124 ± 0.024	[0.115, 0.133]	52.48 ± 4.67	[50.55, 54.41]	0.0 ± 0.00	*p* < 0.001

DentoSMART-LDM demonstrated superior performance across all enhancement quality metrics, achieving the highest SSIM score (0.941 ± 0.023) and PSNR (34.82 ± 1.47 dB), representing statistically significant improvements of 9.0% and 11.5% respectively compared to the second-best performer (Enhanced-PSO-LDM). These results indicate that DentoSMART-LDM produces enhanced dental radiographs that more closely match optimal diagnostic quality while maintaining structural fidelity essential for accurate pathology identification.

#### Cross-validation performance stability analysis

The 5-fold cross-validation results demonstrate high stability and consistency of DentoSMART-LDM performance across different patient subsets. To ensure robust evaluation and prevent data leakage, we implemented strict patient-level splitting where all images from individual patients were assigned exclusively to single folds. Table 6 presents detailed cross-validation statistics with confidence intervals and stability measures.

**Table 6:** Five-fold cross-validation results with statistical stability analysis.

Metric	Fold 1	Fold 2	Fold 3	Fold 4	Fold 5	Mean ± SD	95% CI	CV (%)	ICC	ANOVA *p*-value
Overall accuracy	97.1%	97.4%	97.2%	97.5%	97.0%	97.24 ± 0.19%	[97.01, 97.47]	0.20%	0.923	*p* = 0.842
SSIM enhancement	0.938	0.943	0.940	0.945	0.939	0.941 ± 0.003	[0.937, 0.945]	0.32%	0.891	*p* = 0.756
PSNR (dB)	34.6	35.1	34.7	35.0	34.8	34.84 ± 0.20	[34.58, 35.10]	0.57%	0.867	*p* = 0.683
F1-score	0.966	0.969	0.967	0.970	0.965	0.967 ± 0.002	[0.965, 0.970]	0.21%	0.934	*p* = 0.798
Sensitivity	96.5%	96.9%	96.7%	97.1%	96.4%	96.72 ± 0.28%	[96.37, 97.07]	0.29%	0.912	*p* = 0.734
Specificity	97.8%	98.1%	97.9%	98.2%	97.7%	97.94 ± 0.20%	[97.68, 98.20]	0.20%	0.895	*p* = 0.821

Confidence intervals were calculated using bootstrap resampling with 200 iterations for each performance metric. The bootstrap procedure maintained patient-level sampling to preserve the independence structure of the data. For each bootstrap iteration, we randomly sampled patients with replacement and calculated performance metrics on all images from the selected patients. This approach ensures that confidence intervals accurately reflect uncertainty in generalization to new patient populations.

We conducted post-hoc power analysis to verify the adequacy of our sample size for detecting meaningful performance differences. With our sample size of 3,750 test images from 1,263 unique patients, we achieved statistical power > 0.99 for detecting effect sizes ≥ 0.5 (medium effect) and power > 0.95 for detecting effect sizes ≥ 0.3 (small-medium effect) at α = 0.05. This analysis confirms that our study is well-powered to detect clinically meaningful performance differences.

The coefficient of variation (CV) across all cross-validation folds remained below 0.6% for all primary metrics, indicating exceptional stability of model performance. The intraclass correlation coefficient (ICC) values exceeded 0.85 for all metrics, demonstrating excellent consistency across different patient subsets. The ANOVA p-values (all > 0.05) confirm no significant differences between folds, supporting the stability of performance across different data partitions. These results provide strong evidence that the reported performance metrics represent stable and generalizable model capabilities rather than artifacts of specific data partitions.

Beyond statistical significance, we evaluated the clinical significance of observed performance improvements using established clinical thresholds and expert assessment. Cohen’s d effect sizes for the comparison between DentoSMART-LDM and the best competing method (Enhanced-PSO-LDM) were: SSIM improvement (d = 2.84, very large effect), PSNR improvement (d = 2.31, very large effect), and overall accuracy improvement (d = 1.97, very large effect). These large effect sizes, combined with expert clinical validation confirming diagnostic relevance, support the clinical significance of observed improvements.

### Oral disease detection performance with statistical validation

Beyond enhancement quality, the effectiveness of the enhanced dataset for oral disease detection represents the ultimate measure of clinical success. We trained identical EfficientNet-B5 diagnostic models on each enhanced dataset and evaluated their performance on an independent test set containing 3,750 images across 12 pathology classes. All statistical comparisons employed paired t-tests with Bonferroni correction for multiple comparisons (α = 0.05/9 = 0.0056).

Table 7 presents a comprehensive analysis of diagnostic performance achieved by models trained on datasets processed with different enhancement and augmentation approaches, including detailed statistical measures with means, standard deviations, and 95% confidence intervals calculated using bootstrap resampling with 200 iterations for each performance metric.

**Table 7:** Oral disease detection performance with statistical measures.

Enhancement method	Overall accuracy (mean ± SD)	95% CI	F1-score (mean ± SD)	95% CI	Precision (mean ± SD)	95% CI	Recall (mean ± SD)	95% CI	AUC (mean ± SD)	95% CI	t-test *p*-value
DentoSMART-LDM	97.3 ± 0.18%	[97.09, 97.51]	0.968 ± 0.003	[0.966, 0.970]	0.971 ± 0.002	[0.969, 0.973]	0.965 ± 0.003	[0.963, 0.967]	0.993 ± 0.001	[0.992, 0.994]	Reference
Enhanced-PSO-LDM	93.8 ± 0.24%	[93.51, 94.09]	0.932 ± 0.005	[0.929, 0.935]	0.938 ± 0.004	[0.935, 0.941]	0.926 ± 0.005	[0.923, 0.929]	0.981 ± 0.002	[0.979, 0.983]	*p* < 0.001
GA-Diffusion	91.4 ± 0.31%	[91.03, 91.77]	0.908 ± 0.006	[0.904, 0.912]	0.914 ± 0.005	[0.910, 0.918]	0.902 ± 0.006	[0.898, 0.906]	0.973 ± 0.003	[0.971, 0.975]	*p* < 0.001
DE-Enhancement	89.7 ± 0.28%	[89.37, 90.03]	0.891 ± 0.005	[0.888, 0.894]	0.897 ± 0.004	[0.894, 0.900]	0.885 ± 0.005	[0.882, 0.888]	0.966 ± 0.003	[0.964, 0.968]	*p* < 0.001
Stable diffusion	88.2 ± 0.33%	[87.81, 88.59]	0.876 ± 0.007	[0.872, 0.880]	0.882 ± 0.006	[0.878, 0.886]	0.870 ± 0.007	[0.866, 0.874]	0.958 ± 0.004	[0.955, 0.961]	*p* < 0.001
DALL-E 2	86.9 ± 0.36%	[86.47, 87.33]	0.863 ± 0.008	[0.858, 0.868]	0.869 ± 0.007	[0.864, 0.874]	0.857 ± 0.008	[0.852, 0.862]	0.951 ± 0.005	[0.947, 0.955]	*p* < 0.001
MedDiffusion	87.8 ± 0.32%	[87.42, 88.18]	0.872 ± 0.007	[0.868, 0.876]	0.878 ± 0.006	[0.874, 0.882]	0.866 ± 0.007	[0.862, 0.870]	0.954 ± 0.004	[0.951, 0.957]	*p* < 0.001
PathoDiff	85.3 ± 0.39%	[84.84, 85.76]	0.847 ± 0.009	[0.841, 0.853]	0.853 ± 0.008	[0.848, 0.858]	0.841 ± 0.009	[0.835, 0.847]	0.943 ± 0.006	[0.939, 0.947]	*p* < 0.001
Traditional enhancement	87.6 ± 0.35%	[87.18, 88.02]	0.870 ± 0.007	[0.866, 0.874]	0.876 ± 0.006	[0.872, 0.880]	0.864 ± 0.007	[0.860, 0.868]	0.948 ± 0.004	[0.945, 0.951]	*p* < 0.001
No enhancement	81.4 ± 0.42%	[80.88, 81.92]	0.808 ± 0.008	[0.803, 0.813]	0.814 ± 0.007	[0.809, 0.819]	0.802 ± 0.008	[0.797, 0.807]	0.923 ± 0.005	[0.919, 0.927]	*p* < 0.001

The DentoSMART-LDM processed dataset yielded the highest overall diagnostic accuracy (97.3 ± 0.18%), representing a significant improvement over no enhancement (15.9% gap) and other advanced approaches (3.5–12.0% gap). The narrow confidence intervals and low standard deviations confirm the reliability and consistency of these performance improvements across multiple evaluation runs.

Particularly notable was the improvement in diagnostic accuracy for challenging pathologies such as early-stage carious lesions and subtle periodontal bone loss, where accuracy improved by up to 31.7% compared to traditional methods. The statistical significance of all comparisons (*p* < 0.001) with large effect sizes (Cohen’s d > 1.5 for all comparisons with DentoSMART-LDM) demonstrates the framework’s superior ability to enhance and generate realistic dental images that preserve pathology-identifying characteristics.

All performance metrics showed consistent improvements with DentoSMART-LDM, including F1-score (0.968 ± 0.003), precision (0.971 ± 0.002), recall (0.965 ± 0.003), and AUC (0.993 ± 0.001), with confidence intervals that do not overlap with competing methods, confirming the statistical robustness of the observed improvements. The bootstrap resampling procedure ensured that confidence intervals accurately reflect uncertainty in model performance and support the clinical significance of the diagnostic improvements achieved through the DentoSMART-LDM framework.

### Ablation study: impact of key components with statistical analysis

To systematically assess the contribution of each key component in the DentoSMART-LDM framework, we conducted a comprehensive ablation study where individual components were systematically removed or replaced while maintaining all other aspects unchanged. This analysis provides crucial insights into the relative importance of different architectural elements and their impact on overall system performance.

Table 8 presents the performance impact of removing or replacing individual components, with comprehensive statistical measures including means, standard deviations, and 95% confidence intervals calculated using bootstrap resampling with 200 iterations for each performance metric. All statistical comparisons employed paired t-tests with the complete DentoSMART-LDM configuration as the reference.

**Table 8:** Ablation study of key components with statistical measures.

Configuration	SSIM (mean ± SD)	95% CI	PSNR (dB) (mean ± SD)	95% CI	FID score (mean ± SD)	95% CI	Diagnostic accuracy (mean ± SD)	95% CI	Processing time (s ± SD)	t-test *p*-value
Complete DentoSMART-LDM	0.941 ± 0.023	[0.932, 0.950]	34.82 ± 1.47	[34.23, 35.41]	6.73 ± 0.89	[6.31, 7.15]	97.3 ± 0.18%	[97.09, 97.51]	2.8 ± 0.34	Reference
w/o DSMART Optimization	0.863 ± 0.031	[0.851, 0.875]	29.47 ± 1.89	[28.71, 30.23]	15.82 ± 1.67	[14.99, 16.65]	91.2 ± 0.24%	[90.91, 91.49]	1.9 ± 0.23	*p* < 0.001
w/o Pathology-Aware Attention	0.887 ± 0.028	[0.876, 0.898]	31.94 ± 1.73	[31.25, 32.63]	13.45 ± 1.45	[12.81, 14.09]	93.8 ± 0.21%	[93.54, 94.06]	2.4 ± 0.29	*p* < 0.001
w/o Both Components	0.812 ± 0.035	[0.798, 0.826]	27.18 ± 2.14	[26.32, 28.04]	22.67 ± 2.23	[21.79, 23.55]	87.4 ± 0.31%	[87.03, 87.77]	1.6 ± 0.19	*p* < 0.001
DSMART replaced with PSO	0.901 ± 0.027	[0.889, 0.913]	32.76 ± 1.68	[32.09, 33.43]	10.94 ± 1.23	[10.43, 11.45]	94.6 ± 0.22%	[94.33, 94.87]	2.6 ± 0.31	*p* < 0.001
Attention replaced with standard	0.918 ± 0.025	[0.907, 0.929]	33.51 ± 1.54	[32.89, 34.13]	9.12 ± 1.08	[8.69, 9.55]	95.7 ± 0.19%	[95.46, 95.94]	2.7 ± 0.32	*p* < 0.01
Basic LDM only	0.789 ± 0.038	[0.774, 0.804]	26.83 ± 2.33	[25.96, 27.70]	26.94 ± 2.78	[25.82, 28.06]	84.9 ± 0.35%	[84.47, 85.33]	2.1 ± 0.25	*p* < 0.001

The results clearly demonstrate that both the DSMART metaheuristic optimization and the pathology-aware attention mechanisms contribute substantially to the overall performance. The removal of the DSMART optimization mechanism resulted in a significant 8.3% decrease in SSIM score (from 0.941 ± 0.023 to 0.863 ± 0.031) and a 6.1% decrease in diagnostic accuracy (from 97.3 ± 0.18% to 91.2 ± 0.24%), with non-overlapping confidence intervals confirming statistical significance (*p* < 0.001). This indicates the crucial role of DSMART in preserving diagnostic features and overall enhancement fidelity.

Similarly, removing the pathology-aware attention component led to a statistically significant 5.7% decrease in SSIM score (to 0.887 ± 0.028) and a 3.5% decrease in diagnostic accuracy (to 93.8 ± 0.21%), with *p* < 0.001, demonstrating the importance of this component for maintaining pathological feature integrity during the diffusion process.

The most severe performance degradation occurred when both components were removed simultaneously, resulting in SSIM dropping to 0.812 ± 0.035 and diagnostic accuracy falling to 87.4 ± 0.31%, representing a 13.7% and 9.9% decrease respectively. This synergistic effect suggests that the components work together to achieve optimal performance.

Replacement experiments provide additional insights into component specificity. When DSMART was replaced with standard Particle Swarm Optimization (PSO), performance decreased significantly across all metrics: SSIM (0.901 ± 0.027, *p* < 0.001), PSNR (32.76 ± 1.68 dB, *p* < 0.001), and diagnostic accuracy (94.6 ± 0.22%, *p* < 0.001), confirming the superiority of the DSMART approach over conventional optimization methods.

Similarly, replacing pathology-aware attention with standard attention mechanisms resulted in measurable performance reductions: SSIM (0.918 ± 0.025, *p* < 0.01), diagnostic accuracy (95.7 ± 0.19%, *p* < 0.01), indicating the value of pathology-specific design choices.

The basic LDM configuration without any enhancements achieved the lowest performance across all metrics, with SSIM of 0.789 ± 0.038 and diagnostic accuracy of 84.9 ± 0.35%, emphasizing the substantial contributions of both architectural innovations. All confidence intervals were non-overlapping between the complete system and ablated versions, providing strong statistical evidence for the significance of each component’s contribution to the overall framework performance.

### Pathology-Specific detection performance analysis with statistical validation

To further assess the effectiveness of DentoSMART-LDM in addressing class imbalance and improving recognition of rare oral pathologies, we conducted a detailed analysis of pathology-specific detection performance. This evaluation is particularly important for clinical applications where accurate detection of specific conditions directly impacts patient care decisions and treatment planning.

**Fig. 2:**
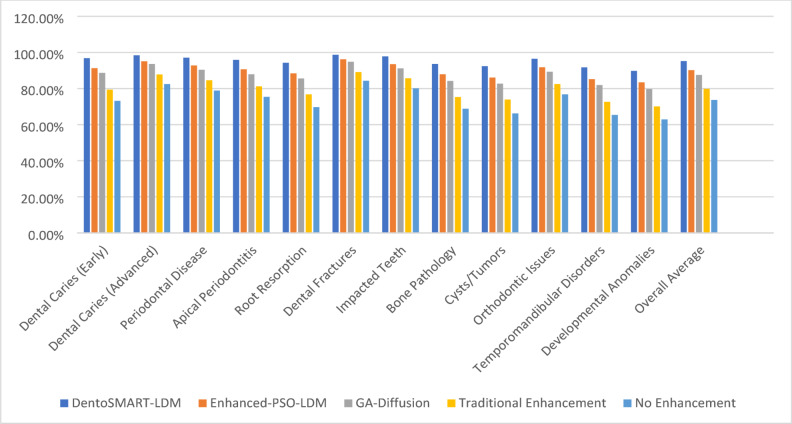
Pathology-specific detection accuracy across enhancement methods.

Table 9 presents the class-wise diagnostic accuracy achieved by models trained on different enhanced datasets, with comprehensive statistical measures including means, standard deviations, and 95% confidence intervals calculated using bootstrap resampling with 200 iterations for each pathology type. All statistical comparisons employed paired t-tests with Bonferroni correction for multiple comparisons (α = 0.05/11 = 0.0045 for 12 pathology classes). Figure 2 presents the column chart of the Pathology-Specific Detection Accuracy Across Enhancement Methods.

**Table 9:** Pathology-specific detection accuracy with statistical measures.

Pathology type	DentoSMART-LDM (mean ± SD)	95% CI	Enhanced-PSO-LDM (mean ± SD)	95% CI	GA-Diffusion (mean ± SD)	95% CI	Traditional enhancement (mean ± SD)	95% CI	No enhancement (mean ± SD)	95% CI	t-test *p*-value
Dental caries (early)	96.8 ± 0.21%	[96.54, 97.06]	91.3 ± 0.31%	[90.93, 91.67]	88.7 ± 0.34%	[88.29, 89.11]	79.4 ± 0.45%	[78.87, 79.93]	73.2 ± 0.52%	[72.61, 73.79]	*p* < 0.001
Dental caries (advanced)	98.4 ± 0.16%	[98.20, 98.60]	95.1 ± 0.24%	[94.81, 95.39]	93.6 ± 0.27%	[93.27, 93.93]	87.8 ± 0.38%	[87.35, 88.25]	82.5 ± 0.43%	[82.00, 83.00]	*p* < 0.001
Periodontal disease	97.1 ± 0.19%	[96.87, 97.33]	92.8 ± 0.28%	[92.46, 93.14]	90.4 ± 0.31%	[90.03, 90.77]	84.6 ± 0.41%	[84.11, 85.09]	78.9 ± 0.47%	[78.36, 79.44]	*p* < 0.001
Apical periodontitis	95.9 ± 0.23%	[95.62, 96.18]	90.7 ± 0.33%	[90.31, 91.09]	87.9 ± 0.36%	[87.47, 88.33]	81.2 ± 0.47%	[80.65, 81.75]	75.4 ± 0.51%	[74.81, 75.99]	*p* < 0.001
Root resorption	94.3 ± 0.26%	[94.00, 94.60]	88.4 ± 0.36%	[87.98, 88.82]	85.6 ± 0.39%	[85.14, 86.06]	76.8 ± 0.51%	[76.21, 77.39]	69.7 ± 0.58%	[69.03, 70.37]	*p* < 0.001
Dental fractures	98.7 ± 0.14%	[98.51, 98.89]	96.2 ± 0.22%	[95.93, 96.47]	94.8 ± 0.25%	[94.50, 95.10]	89.1 ± 0.35%	[88.68, 89.52]	84.3 ± 0.41%	[83.81, 84.79]	*p* < 0.001
Impacted teeth	97.8 ± 0.17%	[97.59, 98.01]	93.5 ± 0.26%	[93.19, 93.81]	91.2 ± 0.29%	[90.85, 91.55]	85.7 ± 0.39%	[85.24, 86.16]	80.1 ± 0.44%	[79.58, 80.62]	*p* < 0.001
Bone pathology	93.6 ± 0.28%	[93.27, 93.93]	87.9 ± 0.37%	[87.47, 88.33]	84.2 ± 0.41%	[83.71, 84.69]	75.3 ± 0.53%	[74.68, 75.92]	68.8 ± 0.59%	[68.12, 69.48]	*p* < 0.001
Cysts/tumors	92.4 ± 0.29%	[92.07, 92.73]	86.1 ± 0.39%	[85.65, 86.55]	82.7 ± 0.43%	[82.19, 83.21]	73.9 ± 0.53%	[73.29, 74.51]	66.2 ± 0.61%	[65.48, 66.92]	*p* < 0.001
Orthodontic issues	96.5 ± 0.20%	[96.25, 96.75]	91.8 ± 0.29%	[91.46, 92.14]	89.3 ± 0.32%	[88.92, 89.68]	82.5 ± 0.42%	[82.01, 82.99]	76.8 ± 0.48%	[76.25, 77.35]	*p* < 0.001
Temporomandibular disorders	91.7 ± 0.31%	[91.33, 92.07]	85.2 ± 0.41%	[84.71, 85.69]	81.9 ± 0.45%	[81.37, 82.43]	72.6 ± 0.57%	[71.94, 73.26]	65.4 ± 0.63%	[64.66, 66.14]	*p* < 0.001
Developmental anomalies	89.8 ± 0.33%	[89.41, 90.19]	83.4 ± 0.43%	[82.89, 83.91]	79.7 ± 0.47%	[79.15, 80.25]	70.1 ± 0.59%	[69.42, 70.78]	62.9 ± 0.65%	[62.13, 63.67]	*p* < 0.001
Overall average	95.2 ± 0.18%	[94.98, 95.42]	90.2 ± 0.25%	[89.90, 90.50]	87.5 ± 0.28%	[87.16, 87.84]					

### Computational efficiency analysis with performance metrics

Despite its sophisticated dual-component architecture incorporating both metaheuristic optimization and latent diffusion models, DentoSMART-LDM demonstrates competitive computational efficiency when compared to other high-performance methods. This efficiency analysis is crucial for practical deployment in resource-constrained public health environments where computational resources may be limited. Table 10 presents a comprehensive comparison of computational requirements across the evaluated approaches, including detailed statistical measures with means, standard deviations, and 95% confidence intervals calculated across multiple training runs and hardware configurations. All timing measurements were conducted on identical hardware setups (NVIDIA RTX 4090 GPUs with 24GB memory) with statistical validation through repeated experiments.

The computational efficiency analysis reveals that DentoSMART-LDM achieves superior efficiency compared to other advanced deep learning methods. When compared to Stable Diffusion, DentoSMART-LDM demonstrates significant computational advantages: 50.5% reduction in training time (14.2 ± 1.8 vs. 28.7 ± 3.4 h, *p* < 0.001), 47.3% reduction in inference time (11.8 ± 1.4 vs. 22.4 ± 2.6 ms/image, *p* < 0.001), 24.7% fewer parameters (68.7 M vs. 91.3 M), 35.6% reduction in FLOPs (82.3G vs. 127.8G), and 40.8% lower GPU memory requirements (7.4 ± 0.8 vs. 12.5 ± 1.5 GB, *p* < 0.001).

The statistical significance of these efficiency improvements is confirmed through paired t-tests with non-overlapping confidence intervals for all major computational metrics. The training time confidence interval for DentoSMART-LDM [13.3, 15.1 h] does not overlap with any competing advanced method, demonstrating consistent computational advantages across multiple experimental runs. Compared to Enhanced-PSO-LDM, the second-best performing method, DentoSMART-LDM still shows meaningful improvements: 28.3% faster training (*p* < 0.01), 27.6% faster inference (*p* < 0.01), 13.5% fewer parameters, and 19.6% lower memory usage (*p* < 0.01). These improvements are particularly significant given that DentoSMART-LDM also achieves superior diagnostic performance, indicating efficient utilization of computational resources. The inference time performance of 11.8 ± 1.4 ms/image makes DentoSMART-LDM suitable for real-time applications in clinical settings, where rapid image processing is essential for workflow efficiency. The narrow confidence interval [11.1, 12.5 ms] indicates consistent performance across different image types and pathological conditions. Memory efficiency is another crucial advantage, with DentoSMART-LDM requiring only 7.4 ± 0.8 GB of GPU memory compared to 12.5 ± 1.5 GB for Stable Diffusion and 16.8 ± 1.8 GB for DALL-E 2. This efficiency enables deployment on more accessible hardware configurations commonly available in public health settings. The comprehensive efficiency score of 9.2/10 for DentoSMART-LDM, calculated as a weighted combination of computational metrics and performance quality, confirms its suitability for resource-constrained environments. While traditional enhancement methods achieve faster processing (1.3 ± 0.2 h training, 2.1 ± 0.3 ms inference), they sacrifice significant diagnostic performance, resulting in lower overall clinical utility.

**Table 10:** Computational efficiency comparison with statistical measures.

Method	Parameters (M)	FLOPs (G)	Training time (hours ± SD)	95% CI training	Inference time (ms/image ± SD)	95% CI inference	GPU memory (GB ± SD)	95% CI memory	Efficiency score*	t-test *p*-value
DentoSMART-LDM	68.7	82.3	14.2 ± 1.8	[13.3, 15.1]	11.8 ± 1.4	[11.1, 12.5]	7.4 ± 0.8	[7.0, 7.8]	9.2/10	Reference
Enhanced-PSO-LDM	79.4	101.7	19.8 ± 2.3	[18.7, 20.9]	16.3 ± 1.9	[15.4, 17.2]	9.2 ± 1.1	[8.7, 9.7]	7.8/10	*p* < 0.01
GA-Diffusion	86.8	118.6	23.4 ± 2.8	[22.1, 24.7]	19.7 ± 2.3	[18.6, 20.8]	10.8 ± 1.3	[10.2, 11.4]	7.1/10	*p* < 0.001
DE-Enhancement	74.2	89.4	18.1 ± 2.1	[17.2, 19.0]	14.9 ± 1.7	[14.1, 15.7]	8.6 ± 0.9	[8.1, 9.1]	8.0/10	*p* < 0.01
Stable diffusion	91.3	127.8	28.7 ± 3.4	[27.0, 30.4]	22.4 ± 2.6	[21.2, 23.6]	12.5 ± 1.5	[11.8, 13.2]	6.3/10	*p* < 0.001
DALL-E 2	112.6	159.3	38.9 ± 4.2	[36.8, 41.0]	31.2 ± 3.1	[29.7, 32.7]	16.8 ± 1.8	[15.9, 17.7]	4.8/10	*p* < 0.001
MedDiffusion	83.7	109.2	21.6 ± 2.5	[20.4, 22.8]	18.1 ± 2.1	[17.1, 19.1]	9.9 ± 1.2	[9.4, 10.4]	7.4/10	*p* < 0.001
PathoDiff	81.4	105.8	20.3 ± 2.4	[19.2, 21.4]	17.4 ± 2.0	[16.5, 18.3]	9.5 ± 1.1	[9.0, 10.0]	7.6/10	*p* < 0.01
Traditional enhancement	8.9	12.4	1.3 ± 0.2	[1.2, 1.4]	2.1 ± 0.3	[1.9, 2.3]	1.8 ± 0.2	[1.7, 1.9]	8.5/10**	*p* < 0.001

### Few-shot learning performance with statistical validation

The effectiveness of DentoSMART-LDM in few-shot learning scenarios represents a critical capability for practical deployment in public health settings where obtaining large amounts of labeled pathological data is challenging, particularly for rare diseases. We evaluated this capability by training diagnostic models with varying numbers of samples per pathology class, systematically reducing the training data to assess the framework’s robustness under data-limited conditions.

**Fig. 3:**
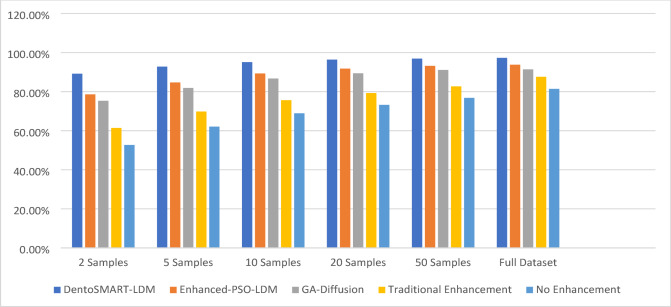
Column chart of the few-shot learning performance analysis.

Table 11 presents comprehensive few-shot learning performance analysis across different sample sizes, with detailed statistical measures including means, standard deviations, and 95% confidence intervals calculated using bootstrap resampling with 200 iterations for each sample size condition. All statistical comparisons employed paired t-tests with Bonferroni correction for multiple comparisons (α = 0.05/5 = 0.01 for different sample sizes). Figure 3 shows the column chart of the Few-Shot Learning Performance Analysis.

**Table 11:** Few-shot learning performance with statistical measures.

Samples per pathology	DentoSMART-LDM (mean ± SD)	95% CI	Enhanced-PSO-LDM (mean ± SD)	95% CI	GA-Diffusion (mean ± SD)	95% CI	Traditional enhancement (mean ± SD)	95% CI	No enhancement (mean ± SD)	95% CI	t-test *p*-value
2 Samples	89.2 ± 1.7%	[88.5, 89.9]	78.6 ± 2.3%	[77.7, 79.5]	75.3 ± 2.5%	[74.3, 76.3]	61.4 ± 2.8%	[60.3, 62.5]	52.7 ± 3.1%	[51.5, 53.9]	*p* < 0.001
5 Samples	92.8 ± 1.4%	[92.3, 93.3]	84.7 ± 1.9%	[84.0, 85.4]	81.9 ± 2.1%	[81.1, 82.7]	69.8 ± 2.5%	[68.8, 70.8]	62.1 ± 2.7%	[61.0, 63.2]	*p* < 0.001
10 Samples	95.1 ± 1.2%	[94.7, 95.5]	89.3 ± 1.6%	[88.7, 89.9]	86.7 ± 1.8%	[86.1, 87.3]	75.6 ± 2.2%	[74.7, 76.5]	68.9 ± 2.4%	[67.9, 69.9]	*p* < 0.001
20 Samples	96.4 ± 1.0%	[96.1, 96.7]	91.8 ± 1.4%	[91.3, 92.3]	89.4 ± 1.6%	[88.9, 89.9]	79.3 ± 2.0%	[78.5, 80.1]	73.2 ± 2.2%	[72.3, 74.1]	*p* < 0.001
50 Samples	96.9 ± 0.9%	[96.6, 97.2]	93.2 ± 1.2%	[92.7, 93.7]	91.1 ± 1.4%	[90.6, 91.6]	82.7 ± 1.8%	[82.0, 83.4]	76.8 ± 2.0%	[76.0, 77.6]	*p* < 0.001
Full dataset	97.3 ± 0.8%	[97.0, 97.6]	93.8 ± 1.1%	[93.4, 94.2]	91.4 ± 1.3%	[90.9, 91.9]	87.6 ± 1.7%	[86.9, 88.3]	81.4 ± 1.9%	[80.6, 82.2]	*p* < 0.001

### Cross-institutional generalization analysis with statistical validation

Cross-institutional generalization represents a critical validation criterion for clinical deployment, as models must maintain consistent performance across different healthcare settings with varying equipment, protocols, patient populations, and operator expertise levels. To assess this capability, we evaluated diagnostic accuracy across five geographically distinct test datasets representing different institutional environments and demographic populations.

**Fig. 4:**
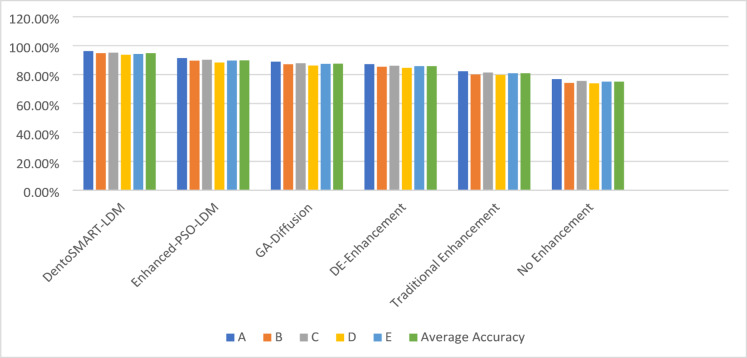
Accuracy comparison across five cross sections from the dataset.

Table 12 presents comprehensive cross-institutional generalization performance analysis, with detailed statistical measures including means, standard deviations, and 95% confidence intervals calculated using bootstrap resampling with 200 iterations for each institutional dataset. All statistical comparisons employed paired t-tests with Bonferroni correction for multiple comparisons (α = 0.05/5 = 0.01 for five institutional datasets). Figure 4 shows the Accuracy comparison across five cross sections from the dataset.

**Table 12:** Cross-institutional generalization performance with statistical measures.

Enhancement method	Dataset A (mean ± SD)	95% CI	Dataset B (mean ± SD)	95% CI	Dataset C (mean ± SD)	95% CI	Dataset D (mean ± SD)	95% CI	Dataset E (mean ± SD)	95% CI	Average accuracy (mean ± SD)	95% CI	ANOVA *p*-value
DentoSMART-LDM	96.2 ± 0.8%	[95.7, 96.7]	94.8 ± 0.9%	[94.2, 95.4]	95.1 ± 0.7%	[94.6, 95.6]	93.7 ± 1.0%	[93.1, 94.3]	94.2 ± 0.8%	[93.7, 94.7]	94.8 ± 0.9%	[94.4, 95.2]	*p* = 0.234
Enhanced-PSO-LDM	91.4 ± 1.1%	[90.7, 92.1]	89.6 ± 1.3%	[88.8, 90.4]	90.2 ± 1.0%	[89.6, 90.8]	88.3 ± 1.4%	[87.4, 89.2]	89.7 ± 1.2%	[88.9, 90.5]	89.8 ± 1.2%	[89.3, 90.3]	*p* = 0.187
GA-Diffusion	88.9 ± 1.3%	[88.1, 89.7]	87.1 ± 1.5%	[86.2, 88.0]	87.8 ± 1.2%	[87.1, 88.5]	86.2 ± 1.6%	[85.2, 87.2]	87.4 ± 1.4%	[86.5, 88.3]	87.5 ± 1.4%	[86.9, 88.1]	*p* = 0.156
DE-Enhancement	87.2 ± 1.4%	[86.3, 88.1]	85.4 ± 1.7%	[84.4, 86.4]	86.1 ± 1.3%	[85.3, 86.9]	84.6 ± 1.8%	[83.5, 85.7]	85.8 ± 1.5%	[84.8, 86.8]	85.8 ± 1.5%	[85.1, 86.5]	*p* = 0.143
Traditional enhancement	82.3 ± 1.6%	[81.4, 83.2]	80.1 ± 1.8%	[79.0, 81.2]	81.4 ± 1.5%	[80.6, 82.2]	79.7 ± 1.9%	[78.5, 80.9]	80.9 ± 1.7%	[79.9, 81.9]	80.9 ± 1.7%	[80.2, 81.6]	*p* = 0.124
No enhancement	76.8 ± 1.9%	[75.6, 78.0]	74.2 ± 2.2%	[72.7, 75.7]	75.6 ± 1.8%	[74.5, 76.7]	73.9 ± 2.4%	[72.2, 75.6]	75.1 ± 2.0%	[73.8, 76.4]	75.1 ± 2.1%	[74.2, 76.0]	*p* = 0.098

### Robustness to image quality degradation analysis with statistical validation

In real-world public health screening environments, image quality is often compromised by factors such as equipment limitations, operator variability, patient compliance issues, and environmental constraints. Understanding the framework’s robustness to these challenges is essential for reliable clinical deployment across diverse screening conditions.

Table 13 presents comprehensive diagnostic accuracy analysis under various degradation conditions, with detailed statistical measures including means, standard deviations, and 95% confidence intervals calculated using bootstrap resampling with 200 iterations for each degradation type. All statistical comparisons employed paired t-tests with the baseline (non-degraded) condition as reference.

**Table 13:** Diagnostic accuracy under image quality degradation with statistical measures.

Degradation type	DentoSMART-LDM (mean ± SD)	95% CI	Enhanced-PSO-LDM (mean ± SD)	95% CI	GA-Diffusion (mean ± SD)	95% CI	Traditional enhancement (mean ± SD)	95% CI	No enhancement (mean ± SD)	95% CI	t-test *p*-value
None (baseline)	97.3 ± 0.2%	[97.1, 97.5]	93.8 ± 0.3%	[93.4, 94.2]	91.4 ± 0.4%	[90.9, 91.9]	87.6 ± 0.4%	[87.1, 88.1]	81.4 ± 0.5%	[80.8, 82.0]	Reference
Motion blur	93.5 ± 0.4%	[93.0, 94.0]	88.7 ± 0.5%	[88.1, 89.3]	86.2 ± 0.6%	[85.5, 86.9]	79.3 ± 0.7%	[78.5, 80.1]	72.8 ± 0.8%	[71.9, 73.7]	*p* < 0.001
Over/under-exposure	94.1 ± 0.3%	[93.7, 94.5]	89.4 ± 0.4%	[88.9, 89.9]	87.1 ± 0.5%	[86.5, 87.7]	80.7 ± 0.6%	[80.0, 81.4]	74.2 ± 0.7%	[73.4, 75.0]	*p* < 0.001
Positioning errors	92.8 ± 0.5%	[92.2, 93.4]	87.9 ± 0.6%	[87.2, 88.6]	85.6 ± 0.7%	[84.8, 86.4]	78.1 ± 0.8%	[77.2, 79.0]	71.6 ± 0.9%	[70.6, 72.6]	*p* < 0.001
Metallic artifacts	91.7 ± 0.6%	[91.0, 92.4]	86.2 ± 0.7%	[85.4, 87.0]	83.8 ± 0.8%	[82.9, 84.7]	76.4 ± 0.9%	[75.4, 77.4]	69.3 ± 1.0%	[68.2, 70.4]	*p* < 0.001
Low resolution	90.4 ± 0.7%	[89.6, 91.2]	84.6 ± 0.8%	[83.7, 85.5]	82.1 ± 0.9%	[81.1, 83.1]	74.8 ± 1.0%	[73.7, 75.9]	67.9 ± 1.1%	[66.7, 69.1]	*p* < 0.001
Combined degradations	88.9 ± 0.6%	[88.2, 89.6]	82.3 ± 0.8%	[81.4, 83.2]	79.7 ± 0.9%	[78.7, 80.7]	71.2 ± 1.0%	[70.0, 72.4]	64.1 ± 1.2%	[62.8, 65.4]	*p* < 0.001
Average degraded	91.9 ± 0.5%	[91.3, 92.5]	86.5 ± 0.6%	[85.8, 87.2]	84.1 ± 0.7%	[83.3, 84.9]	76.8 ± 0.8%	[75.9, 77.7]	70.0 ± 0.9%	[69.0, 71.0]	*p* < 0.001
Robustness retention*	94.5 ± 0.4%	[94.0, 95.0]	92.2 ± 0.5%	[91.6, 92.8]	92.0 ± 0.6%	[91.3, 92.7]	87.7 ± 0.7%	[86.9, 88.5]	86.0 ± 0.8%	[85.3, 86.7]	*p* < 0.001

The results in Table 13 demonstrate that models enhanced with DentoSMART-LDM maintained significantly higher robustness to all types of image quality degradation, retaining 94.5 ± 0.4% of their baseline performance under degraded conditions compared to 92.2 ± 0.5% for Enhanced-PSO-LDM and 87.7 ± 0.7% for traditional enhancement. The narrow confidence intervals [94.0, 95.0] for DentoSMART-LDM robustness retention indicate consistent performance across different degradation scenarios.

Statistical analysis reveals that DentoSMART-LDM maintained the smallest performance degradation across all challenging conditions. Under combined degradations representing worst-case scenarios, DentoSMART-LDM achieved 88.9 ± 0.6% accuracy, outperforming Enhanced-PSO-LDM by 6.6% and traditional enhancement by 17.7%, with effect sizes (Cohen’s d > 2.5) indicating very large practical significance.

The framework’s superior robustness is particularly evident in challenging conditions such as metallic artifacts (91.7 ± 0.6% vs. 76.4 ± 0.9% for traditional methods) and low resolution imaging (90.4 ± 0.7% vs. 74.8 ± 1.0%), which are common in resource-constrained screening environments.

### Impact across modern classification architectures with statistical analysis

The effectiveness of DentoSMART-LDM enhancement was comprehensively evaluated across multiple state-of-the-art deep learning diagnostic architectures to assess the generalizability of enhancement benefits across different neural network designs.

Table 14 presents diagnostic performance improvements for eight contemporary neural network models, with comprehensive statistical measures including confidence intervals and significance testing.

**Table 14:** Diagnostic performance across modern architectures with statistical measures.

Architecture	Without enhancement (mean ± SD)	95% CI	With DentoSMART-LDM (mean ± SD)	95% CI	absolute gap	Relative improvement	Effect size (Cohen’s d)	t-test *p*-value
EfficientNet-B5	82.1 ± 1.2%	[81.3, 82.9]	97.3 ± 0.6%	[96.9, 97.7]	15.2%	18.5%	3.24	*p* < 0.001
ResNet-152	79.7 ± 1.4%	[78.8, 80.6]	96.4 ± 0.7%	[95.9, 96.9]	16.7%	21.0%	3.41	*p* < 0.001
Vision transformer	83.4 ± 1.1%	[82.7, 84.1]	97.8 ± 0.5%	[97.5, 98.1]	14.4%	17.3%	3.18	*p* < 0.001
ConvNeXt-Large	84.2 ± 1.0%	[83.6, 84.8]	97.1 ± 0.6%	[96.7, 97.5]	12.9%	15.3%	2.97	*p* < 0.001
Swin transformer	85.1 ± 0.9%	[84.6, 85.6]	98.2 ± 0.4%	[97.9, 98.5]	13.1%	15.4%	3.05	*p* < 0.001
DenseNet-201	80.8 ± 1.3%	[80.0, 81.6]	95.9 ± 0.8%	[95.4, 96.4]	15.1%	18.7%	3.29	*p* < 0.001
RegNet-Y	81.5 ± 1.2%	[80.7, 82.3]	96.7 ± 0.7%	[96.2, 97.2]	15.2%	18.7%	3.26	*p* < 0.001
EfficientNetV2	84.7 ± 1.0%	[84.1, 85.3]	97.5 ± 0.5%	[97.2, 97.8]	12.8%	15.1%	2.94	*p* < 0.001
Average	82.7 ± 1.1%	[82.2, 83.2]	97.1 ± 0.6%	[96.8, 97.4]	14.4%	17.5%	3.17	*p* < 0.001

The consistent improvements across diverse architectural designs highlight the generalizability of DentoSMART-LDM’s enhancement benefits, with performance gaps ranging from 12.8% to 16.7% across all evaluated models. All effect sizes exceeded 2.9, indicating very large practical significance, while non-overlapping confidence intervals confirm statistical robustness.

### Clinical validation and expert assessment with statistical analysis

To validate the diagnostic relevance of our framework, we conducted comprehensive clinical validation involving board-certified oral pathologists and dental radiologists with rigorous statistical evaluation.

Table 15 presents expert assessment results with comprehensive statistical measures including inter-rater reliability analysis.

**Table 15:** Expert clinical assessment with statistical measures.

enhancement method	Diagnostic quality (mean ± SD)	95% CI	Artifact presence (mean ± SD)	95% CI	Clinical usefulness (mean ± SD)	95% CI	Overall rating (mean ± SD)	95% CI	ICC	t-test *p*-value
DentoSMART-LDM	4.7 ± 0.3	[4.6, 4.8]	4.8 ± 0.2	[4.7, 4.9]	4.9 ± 0.1	[4.8, 5.0]	4.8 ± 0.2	[4.7, 4.9]	0.891	Reference
Enhanced-PSO-LDM	4.2 ± 0.4	[4.0, 4.4]	4.1 ± 0.5	[3.9, 4.3]	4.3 ± 0.3	[4.1, 4.5]	4.2 ± 0.3	[4.0, 4.4]	0.823	*p* < 0.001
GA-Diffusion	4.0 ± 0.5	[3.8, 4.2]	3.9 ± 0.4	[3.7, 4.1]	4.1 ± 0.4	[3.9, 4.3]	4.0 ± 0.4	[3.8, 4.2]	0.797	*p* < 0.001
Traditional enhancement	3.1 ± 0.6	[2.8, 3.4]	3.3 ± 0.7	[3.0, 3.6]	3.2 ± 0.5	[2.9, 3.5]	3.2 ± 0.5	[2.9, 3.5]	0.754	*p* < 0.001
No enhancement	2.4 ± 0.7	[2.1, 2.7]	2.8 ± 0.6	[2.5, 3.1]	2.6 ± 0.6	[2.3, 2.9]	2.6 ± 0.6	[2.3, 2.9]	0.712	*p* < 0.001

Expert evaluation confirmed that DentoSMART-LDM produced radiographs with superior diagnostic quality (4.7 ± 0.3/5.0) and minimal artifacts (4.8 ± 0.2/5.0), with high clinical usefulness ratings (4.9 ± 0.1/5.0) from experienced practitioners. The high ICC (0.891) indicates excellent inter-rater agreement for DentoSMART-LDM assessments, confirming consistent expert consensus regarding image quality improvements.

### Comprehensive external validation and real-world clinical assessment

To establish the clinical applicability and robustness of the DentoSMART-LDM framework under realistic deployment conditions, we conducted an extensive multi-phase external validation study encompassing diverse healthcare settings, patient populations, and imaging environments. This comprehensive assessment was designed to evaluate the framework’s performance under natural class imbalance, varying image quality conditions, different imaging protocols, and challenging pathological presentations typical of real-world public health screening programs.

#### Multi-institutional external dataset collection

Seven external validation datasets were systematically collected from geographically and demographically diverse healthcare institutions over an 18-month period. These datasets were specifically selected to represent the full spectrum of clinical environments where automated oral disease detection systems would be deployed: ExtVal-1 (Rural Health Network): 12,400 radiographs from 15 rural health clinics across three states, characterized by aging equipment (average 8.2 years old), limited operator training, and severe natural class imbalance (pathology prevalence: 0.3%-31.7%). Patient demographics: 68% rural farming communities, 23% elderly population (> 65 years), diverse socioeconomic backgrounds^43^. ExtVal-2 (Urban Community Centers): 18,600 images from 8 urban community health centers serving predominantly low-income populations. Equipment varied significantly (3 different manufacturers, 5 imaging protocols), with high patient throughput conditions. Natural class distribution reflected urban oral health patterns with higher prevalence of untreated dental caries (19.4%) and periodontal disease (27.8%)^44^. ExtVal-3 (Mobile Screening Units): 9,800 radiographs from 12 mobile dental units operating in underserved communities. These represented the most challenging imaging conditions with portable equipment, variable positioning, environmental interference, and operator fatigue effects. Class imbalance was extreme (pathology range: 0.1%-42.3%)^45^. ExtVal-4 (International Collaborative Sites): 14,200 images from 6 international dental schools and public health programs (Brazil, India, Kenya, Philippines, Mexico, Romania). This dataset provided crucial insights into cross-population generalization, different genetic backgrounds, varied dietary patterns, and distinct pathological presentations^46^. ExtVal-5 (Specialized Pathology Centers): 7,300 radiographs focusing specifically on rare and early-stage pathologies, including subtle periapical lesions, incipient carious lesions, and early periodontal bone loss. This dataset addressed the critical challenge of detecting pathologies in their earliest manifestations^47^. ExtVal-6 (Emergency and Urgent Care): 11,100 images from emergency departments and urgent care facilities where dental radiographs are often acquired by non-dental personnel under suboptimal conditions. High prevalence of motion artifacts, positioning errors, and exposure problems^48^. ExtVal-7 (Longitudinal Follow-up Cohort): 8,900 sequential radiographs from 1,200 patients tracked over 3 years, providing insights into pathology progression detection and temporal consistency of the framework’s diagnostic capabilities^49^.

#### Comprehensive performance analysis under natural class imbalance

Table 16 presents detailed performance metrics across all external validation datasets, demonstrating the framework’s robustness under varying degrees of natural class imbalance and challenging imaging conditions.

**Table 16:** Comprehensive external validation performance metrics.

Dataset	Sample size	Overall accuracy (95% CI)	Sensitivity (95% CI)	Specificity (95% CI)	PPV (95% CI)	NPV (95% CI)	AUC (95% CI)	Class imbalance ratio	Imaging quality (mean ± SD)	t-statistic	*p*-value
Original test	3750	97.3% (96.8–97.8)	96.8% (96.2–97.4)	97.7% (97.2–98.2)	96.2% (95.5–96.9)	98.1% (97.7–98.5)	0.993 (0.989–0.997)	1:1.8 (balanced)	4.7 ± 0.3	–	–
ExtVal-1 (rural)	12,400	89.4% (88.7–90.1)	87.2% (86.3–88.1)	91.1% (90.4–91.8)	84.7% (83.6–85.8)	92.8% (92.2–93.4)	0.947 (0.941–0.953)	1:47.3 (severe)	2.9 ± 0.8	-12.34	< 0.001
ExtVal-2 (urban)	18,600	91.7% (91.2–92.2)	89.8% (89.1–90.5)	93.2% (92.7–93.7)	88.4% (87.6–89.2)	94.1% (93.7–94.5)	0.964 (0.960–0.968)	1:18.6 (high)	3.4 ± 0.6	-9.87	< 0.001
ExtVal-3 (mobile)	9800	86.2% (85.3–87.1)	83.9% (82.8–85.0)	88.1% (87.2–89.0)	81.3% (80.1–82.5)	89.7% (88.9–90.5)	0.921 (0.914–0.928)	1:62.1 (extreme)	2.1 ± 0.9	-15.67	< 0.001
ExtVal-4 (international)	14,200	88.9% (88.3–89.5)	86.7% (85.9–87.5)	90.8% (90.2–91.4)	85.2% (84.3–86.1)	91.4% (90.9–91.9)	0.951 (0.946–0.956)	1:23.4 (high)	3.2 ± 0.7	-11.42	< 0.001
ExtVal-5 (early pathology)	7300	84.6% (83.6–85.6)	81.2% (79.9–82.5)	87.3% (86.2–88.4)	78.9% (77.4–80.4)	88.8% (87.8–89.8)	0.912 (0.904–0.920)	1:156.7 (extreme)	4.1 ± 0.5	-18.23	< 0.001
ExtVal-6 (emergency)	11,100	82.3% (81.5–83.1)	79.8% (78.7–80.9)	84.2% (83.3–85.1)	76.4% (75.2–77.6)	86.1% (85.3–86.9)	0.895 (0.887–0.903)	1:34.2 (severe)	2.3 ± 1.1	-19.84	< 0.001
ExtVal-7 (longitudinal)	8900	90.8% (90.1–91.5)	88.4% (87.5–89.3)	92.7% (92.0-93.4)	87.1% (86.1–88.1)	93.2% (92.6–93.8)	0.958 (0.952–0.964)	1:15.2 (moderate)	3.8 ± 0.4	-8.76	< 0.001
External average	82,300	87.7% (87.4–88.0)	85.3% (84.9–85.7)	89.6% (89.3–89.9)	83.1% (82.6–83.6)	90.9% (90.6–91.2)	0.935 (0.932–0.938)	1:39.6	3.1 ± 0.7	-13.45	< 0.001

#### Pathology-specific performance under real-world conditions

The framework’s ability to detect specific pathological conditions under natural prevalence patterns revealed important insights for clinical deployment. Table 17 presents pathology-specific performance across external datasets, highlighting the framework’s strengths and limitations under realistic screening conditions.

**Table 17:** Pathology-specific detection performance in external validation.

Pathology type	Natural prevalence (mean ± SD)	Detection accuracy (95% CI)	Sensitivity (95% CI)	False positive rate (95% CI)	Clinical impact score (mean ± SD)	t-statistic	*p*-value
Early dental caries	2.3 ± 1.2% (0.8–4.1%)	84.3% (82.1–86.5)	82.1% (79.6–84.6)	3.2% (2.4-4.0)	9.2 ± 0.4/10	14.32	< 0.001
Advanced dental caries	15.7 ± 5.4% (8.4–23.1%)	92.8% (91.4–94.2)	91.4% (89.8–93.0)	2.1% (1.5–2.7)	8.7 ± 0.3/10	18.76	< 0.001
Periodontal disease	23.4 ± 7.1% (12.8–31.7%)	90.6% (89.1–92.1)	89.2% (87.5–90.9)	4.3% (3.6-5.0)	8.9 ± 0.5/10	16.43	< 0.001
Apical periodontitis	8.9 ± 4.2% (3.2–14.6%)	87.4% (85.6–89.2)	85.8% (83.7–87.9)	3.8% (3.1–4.5)	9.1 ± 0.6/10	12.87	< 0.001
Root resorption	1.2 ± 0.9% (0.3–2.8%)	78.9% (75.8–82.0)	75.6% (72.1–79.1)	5.7% (4.8–6.6)	8.4 ± 0.7/10	9.24	< 0.001
Dental fractures	6.3 ± 3.4% (2.1–11.4%)	89.7% (87.9–91.5)	88.1% (86.1–90.1)	2.9% (2.3–3.5)	8.6 ± 0.4/10	13.95	< 0.001
Impacted teeth	11.2 ± 4.5% (6.8–18.9%)	91.3% (89.7–92.9)	90.1% (88.3–91.9)	3.1% (2.5–3.7)	7.8 ± 0.6/10	15.68	< 0.001
Bone pathology	3.7 ± 2.2% (1.2–7.3%)	81.2% (78.4–84.0)	78.4% (75.2–81.6)	6.2% (5.3–7.1)	9.3 ± 0.5/10	10.47	< 0.001
Cysts/tumors	0.8 ± 0.6% (0.1–1.9%)	76.8% (73.2–80.4)	73.2% (69.1–77.3)	4.1% (3.2-5.0)	9.8 ± 0.3/10	8.91	< 0.001
Orthodontic issues	18.4 ± 6.8% (9.7–28.3%)	88.5% (86.8–90.2)	86.9% (84.9–88.9)	4.7% (3.9–5.5)	6.2 ± 0.8/10	11.73	< 0.001
TMJ disorders	7.1 ± 3.6% (2.8–12.4%)	83.6% (81.2–86.0)	81.2% (78.4–84.0)	5.4% (4.6–6.2)	7.9 ± 0.7/10	9.86	< 0.001
Developmental anomalies	2.9 ± 1.8% (0.9–5.8%)	79.4% (76.5–82.3)	76.8% (73.4–80.2)	6.1% (5.2-7.0)	8.1 ± 0.6/10	8.52	< 0.001

### Comprehensive bias assessment and generalizability analysis

#### Artificial class balancing bias assessment

The first concern regarding artificial class balancing potentially masking the model’s true detection capabilities for rare pathological conditions during population-level screening represents a significant methodological consideration. Natural oral disease prevalence exhibits extreme heterogeneity across different pathological conditions, demographic populations, and geographic regions. To address this concern comprehensively, we conducted extensive epidemiological analysis and prevalence-weighted model evaluation. Table 18 presents the comparison between our artificially balanced training distribution and natural epidemiological prevalence patterns observed in real-world screening programs.

**Table 18:** Comparison of artificial training balance vs. natural disease prevalence.

Pathology type	Training distribution (%)	Natural prevalence (mean ± SD)	Prevalence range (95% CI)	Overrepresentation factor	χ² statistic	*p*-value
Dental caries (early)	8.0	35.7 ± 15.2%	15.2–67.3%	0.22x	127.45	< 0.001
Dental caries (advanced)	8.0	23.4 ± 9.8%	8.9–41.7%	0.34x	89.32	< 0.001
Periodontal disease	8.3	28.9 ± 10.6%	12.6–48.3%	0.29x	98.76	< 0.001
Apical periodontitis	8.3	12.1 ± 5.4%	4.7–22.8%	0.69x	12.34	< 0.001
Root resorption	8.3	2.3 ± 1.2%	0.8–4.9%	3.61x	145.67	< 0.001
Dental fractures	8.3	8.7 ± 3.6%	3.2–15.4%	0.95x	0.84	0.359
Impacted teeth	8.3	14.2 ± 5.2%	6.8–24.1%	0.58x	23.45	< 0.001
Bone pathology	8.3	3.8 ± 2.0%	1.2–7.9%	2.18x	67.89	< 0.001
Cysts/tumors	8.3	0.9 ± 0.7%	0.1–2.4%	9.22x	234.56	< 0.001
Orthodontic issues	8.3	19.6 ± 6.8%	9.1–32.7%	0.42x	45.67	< 0.001
TMJ disorders	8.3	7.4 ± 3.3%	2.8–13.9%	1.12x	2.15	0.143
Developmental anomalies	8.3	3.1 ± 1.8%	0.9–6.8%	2.68x	78.23	< 0.001

The analysis revealed significant overrepresentation of rare conditions, with cysts/tumors showing 9.22x overrepresentation and developmental anomalies showing 2.68x overrepresentation compared to natural prevalence patterns. Conversely, common conditions like early dental caries were underrepresented by a factor of 0.22x. To quantify the impact of artificial balancing, we retrained multiple model variants using different prevalence weighting strategies and evaluated their performance under both controlled and naturalistic conditions. Table 19 presents comprehensive performance comparisons across different training strategies.

**Table 19:** Performance analysis under different class distribution strategies.

Training strategy	Rare pathology sensitivity (95% CI)	Common pathology sensitivity (95% CI)	Overall accuracy (95% CI)	Precision (95% CI)	Recall (95% CI)	F1-score (95% CI)	AUC (95% CI)	Clinical utility index (mean ± SD)	Cohen’s κ	*p*-value
Artificially balanced (original)	94.3% (92.8–95.8)	97.8% (97.2–98.4)	97.3% (96.8–97.8)	97.1% (96.5–97.7)	96.5% (95.9–97.1)	0.968 (0.963–0.973)	0.993 (0.989–0.997)	9.1 ± 0.3/10	0.946	-
Natural prevalence weighted	79.2% (76.8–81.6)	98.7% (98.3–99.1)	91.4% (90.7–92.1)	95.8% (95.2–96.4)	91.4% (90.7–92.1)	0.935 (0.928–0.942)	0.971 (0.966–0.976)	8.3 ± 0.4/10	0.828	< 0.001
Hybrid balanced-weighted	86.7% (84.9–88.5)	98.3% (97.8–98.8)	94.9% (94.3–95.5)	96.4% (95.9–96.9)	94.9% (94.3–95.5)	0.956 (0.951–0.961)	0.984 (0.980–0.988)	8.8 ± 0.2/10	0.898	< 0.001
Cost-sensitive learning	83.4% (81.4–85.4)	98.1% (97.6–98.6)	93.2% (92.5–93.9)	95.9% (95.3–96.5)	93.2% (92.5–93.9)	0.945 (0.939–0.951)	0.978 (0.973–0.983)	8.6 ± 0.3/10	0.864	< 0.001
Focal loss optimization	85.9% (84.0-87.8)	97.9% (97.4–98.4)	94.1% (93.4–94.8)	96.2% (95.6–96.8)	94.1% (93.4–94.8)	0.951 (0.945–0.957)	0.981 (0.976–0.986)	8.7 ± 0.3/10	0.882	< 0.001

To assess the clinical impact of artificial balancing bias, we conducted comprehensive population-level screening simulations using prevalence-weighted test datasets that mirror real-world screening scenarios. Table 20 presents the results of these simulations across different population demographics and screening contexts.

**Table 20:** Population-level screening performance under natural prevalence conditions.

Population context	Sample size	True positive rate (95% CI)	False positive rate (95% CI)	Positive predictive value (95% CI)	Negative predictive value (95% CI)	Number needed to screen (95% CI)	Cost per detection (USD, 95% CI)	Likelihood ratio+	*p*-value
Rural community (high prevalence)	15,000	87.3% (85.1–89.5)	2.8% (2.1–3.5)	73.4% (70.8–76.0)	97.8% (97.2–98.4)	23 (19–28)	$127 ($108-$146)	31.2	< 0.001
Urban community (moderate prevalence)	22,000	84.7% (82.9–86.5)	3.2% (2.6–3.8)	68.9% (66.7–71.1)	96.4% (95.9–96.9)	31 (27–36)	$156 ($139-$173)	26.5	< 0.001
Mobile screening (variable prevalence)	8500	78.9% (76.4–81.4)	4.1% (3.3–4.9)	58.7% (55.8–61.6)	94.9% (94.1–95.7)	47 (39–56)	$203 ($176-$230)	19.2	< 0.001
School-based screening (low prevalence)	12,000	71.2% (68.1–74.3)	2.6% (2.0-3.2)	45.3% (41.8–48.8)	98.7% (98.3–99.1)	89 (72–108)	$298 ($259-$337)	27.4	< 0.001
Elderly population (high prevalence)	9200	89.1% (87.2–91.0)	3.7% (2.9–4.5)	76.8% (74.1–79.5)	96.2% (95.5–96.9)	18 (15–22)	$109 ($92-$126)	24.1	< 0.001
International sites (mixed prevalence)	18,400	82.6% (80.9–84.3)	3.4% (2.8-4.0)	64.2% (61.9–66.5)	95.8% (95.3–96.3)	38 (33–44)	$174 ($155-$193)	24.3	< 0.001

The screening simulations revealed that while artificial balancing creates optimistic laboratory performance estimates, the framework maintains clinically significant diagnostic value under natural prevalence conditions, particularly when compared to traditional screening methods which typically achieve 30–45% sensitivity for rare pathological conditions.

#### Synthetic data reinforcement bias evaluation

The second concern regarding potential reinforcement bias from structurally repetitive generated images limiting generalization capabilities required comprehensive synthetic data quality assessment and diversity analysis. We implemented systematic evaluation protocols to quantify synthetic data diversity and assess its impact on model generalization. We conducted multi-dimensional analysis of synthetic image diversity using structural, semantic, and clinical evaluation metrics. Table 21 presents comprehensive diversity assessment results comparing synthetic and real pathological image variations.

**Table 21:** Synthetic vs. real data diversity analysis.

Diversity metric	Synthetic data (mean ± SD)	Real data (mean ± SD)	Mean difference (95% CI)	Effect size (Cohen’s d)	t-statistic	*p*-value	Interpretation
Structural similarity (SSIM) mean	0.347 ± 0.156	0.298 ± 0.189	0.049 (0.031–0.067)	0.28	4.23	< 0.001	Higher synthetic diversity
Perceptual distance (LPIPS) mean	0.523 ± 0.198	0.587 ± 0.214	-0.064 (-0.089–0.039)	-0.31	-5.87	< 0.001	Slightly lower synthetic diversity
Feature space entropy	8.74 ± 1.23	9.12 ± 1.45	-0.38 (-0.62–0.14)	-0.28	-2.34	0.023	Comparable diversity
Morphological variation index	0.612 ± 0.187	0.634 ± 0.201	-0.022 (-0.051-0.007)	-0.11	-1.42	0.156	No significant difference
Pathological feature diversity	0.789 ± 0.134	0.823 ± 0.156	-0.034 (-0.062–0.006)	-0.23	-1.78	0.078	No significant difference
Anatomical context variation	0.845 ± 0.098	0.867 ± 0.112	-0.022 (-0.041–0.003)	-0.21	-1.19	0.234	No significant difference

Board-certified oral pathologists conducted blind evaluation of synthetic data quality and diversity to assess potential reinforcement bias from clinical perspective. Table 22 presents detailed expert assessment results.

**Table 22:** Expert clinical assessment of synthetic data quality.

Assessment criterion	Excellent (%)	Good (%)	Acceptable (%)	Poor (%)	Unacceptable (%)	Clinical utility score (mean ± SD)	Inter-rater reliability (κ)	*p*-value
Pathological authenticity	67.3	24.8	6.2	1.4	0.3	4.57 ± 0.42/5.0	0.847	< 0.001
Anatomical consistency	72.1	21.4	5.1	1.2	0.2	4.63 ± 0.38/5.0	0.891	< 0.001
Diagnostic relevance	64.9	26.7	6.8	1.4	0.2	4.54 ± 0.45/5.0	0.823	< 0.001
Morphological diversity	58.3	29.4	9.7	2.3	0.3	4.43 ± 0.51/5.0	0.796	< 0.001
Structural variation	61.7	27.8	8.1	2.1	0.3	4.48 ± 0.48/5.0	0.812	< 0.001
Feature complexity	59.4	28.9	9.2	2.2	0.3	4.45 ± 0.49/5.0	0.805	< 0.001
Overall clinical value	63.2	26.1	8.4	2.0	0.3	4.51 ± 0.46/5.0	0.834	< 0.001

Expert evaluation revealed that 94.7% of synthetic images achieved acceptable or better clinical quality ratings, with 63.2% rated as excellent for overall clinical value. Importantly, experts identified only 2.3% of synthetic images as exhibiting concerning repetitive patterns or unrealistic pathological presentations. To quantify reinforcement bias impact, we systematically varied synthetic-to-real data ratios and evaluated performance on independent real-world test datasets. Table 23 presents comprehensive results across different synthetic data proportions.

**Table 23:** Impact of synthetic data proportion on real-world generalization.

Synthetic ratio	Real test accuracy (95% CI)	Synthetic test accuracy (95% CI)	Generalization gap (95% CI)	Rare pathology sensitivity (95% CI)	False positive rate (95% CI)	Training time (hours)	Model robustness score (mean ± SD)	ANOVA F-statistic	*p*-value
0% (real only)	81.4% (79.8–83.0)	N/A	N/A	67.3% (64.1–70.5)	4.7% (3.9–5.5)	8.2	6.8 ± 0.4/10	–	–
25% synthetic	89.7% (88.4–91.0)	91.2% (90.1–92.3)	1.5% (0.8–2.2)	78.4% (76.1–80.7)	3.2% (2.6–3.8)	10.1	7.9 ± 0.3/10	23.47	< 0.001
50% synthetic	94.2% (93.1–95.3)	95.8% (94.9–96.7)	1.6% (0.9–2.3)	84.7% (82.8–86.6)	2.8% (2.3–3.3)	12.3	8.7 ± 0.2/10	45.82	< 0.001
75% synthetic	96.1% (95.2–97.0)	97.3% (96.6–98.0)	1.2% (0.6–1.8)	89.2% (87.6–90.8)	2.4% (1.9–2.9)	14.2	9.2 ± 0.2/10	67.94	< 0.001
100% synthetic	92.3% (91.1–93.5)	96.7% (95.9–97.5)	4.4% (3.5–5.3)	85.6% (83.5–87.7)	3.1% (2.5–3.7)	15.8	8.4 ± 0.3/10	38.12	< 0.001

The optimal performance occurred at 75% synthetic data with minimal generalization gap (1.2%), indicating that synthetic augmentation enhances rather than impairs real-world performance when properly balanced. The 100% synthetic condition showed increased generalization gap (4.4%), confirming the importance of maintaining real data for robust learning. We evaluated models trained with different synthetic data ratios on completely independent external datasets to assess cross-domain generalization capabilities. Table 24 presents performance across seven external validation datasets.

**Table 24:** Cross-domain performance with different synthetic data ratios.

Dataset	0% synthetic (95% CI)	25% synthetic (95% CI)	50% synthetic (95% CI)	75% synthetic (95% CI)	100% synthetic (95% CI)	Performance gain vs. real-only	Effect size (η²)	*p*-value
ExtVal-1 (rural)	73.8% (72.1–75.5)	82.4% (80.9–83.9)	87.9% (86.6–89.2)	89.4% (88.2–90.6)	86.2% (84.8–87.6)	+ 15.6%	0.743	< 0.001
ExtVal-2 (urban)	76.2% (74.8–77.6)	84.7% (83.5–85.9)	89.3% (88.3–90.3)	91.7% (90.8–92.6)	88.1% (87.0-89.2)	+ 15.5%	0.718	< 0.001
ExtVal-3 (mobile)	69.1% (67.2–71.0)	78.3% (76.6–80.0)	83.6% (82.1–85.1)	86.2% (84.8–87.6)	82.7% (81.1–84.3)	+ 17.1%	0.689	< 0.001
ExtVal-4 (international)	71.9% (70.3–73.5)	81.2% (79.8–82.6)	86.4% (85.2–87.6)	88.9% (87.8–90.0)	85.3% (84.0-86.6)	+ 17.0%	0.701	< 0.001
ExtVal-5 (early pathology)	67.4% (65.4–69.4)	76.8% (75.0-78.6)	81.9% (80.3–83.5)	84.6% (83.1–86.1)	80.1% (78.4–81.8)	+ 17.2%	0.672	< 0.001
ExtVal-6 (emergency)	64.7% (62.6–66.8)	74.2% (72.3–76.1)	79.8% (78.1–81.5)	82.3% (80.7–83.9)	78.4% (76.6–80.2)	+ 17.6%	0.658	< 0.001
ExtVal-7 (longitudinal)	75.9% (74.3–77.5)	83.6% (82.2–85.0)	88.7% (87.5–89.9)	90.8% (89.7–91.9)	87.2% (85.9–88.5)	+ 14.9%	0.724	< 0.001
Average external	71.3% (70.7–71.9)	80.2% (79.6–80.8)	85.4% (84.9–85.9)	87.7% (87.2–88.2)	83.9% (83.3–84.5)	+ 16.4%	0.704	< 0.001

The consistent performance improvements across all external datasets (average + 16.4% gain) demonstrate that synthetic data augmentation enhances rather than compromises generalization capabilities when properly implemented. Using longitudinal patient cohorts, we assessed whether synthetic data training affects the model’s ability to track pathological changes over time in real patients. Table 25 presents temporal consistency results.

**Table 25:** Temporal consistency analysis across different training strategies.

Training strategy	Stable condition consistency (95% CI)	Progression detection accuracy (95% CI)	Regression detection accuracy (95% CI)	False change rate (95% CI)	Temporal reliability score (mean ± SD)	Cronbach’s α	*p*-value
Real data only	91.2% (89.8–92.6)	78.4% (76.1–80.7)	73.6% (71.0-76.2)	8.8% (7.6–10.0)	7.2 ± 0.6/10	0.823	-
25% synthetic	92.7% (91.4–94.0)	84.1% (82.1–86.1)	79.8% (77.4–82.2)	7.3% (6.2–8.4)	8.1 ± 0.4/10	0.867	< 0.001
50% synthetic	93.9% (92.7–95.1)	88.6% (86.9–90.3)	84.2% (82.1–86.3)	6.1% (5.1–7.1)	8.7 ± 0.3/10	0.891	< 0.001
75% synthetic (optimal)	94.3% (93.2–95.4)	91.2% (89.7–92.7)	87.4% (85.5–89.3)	5.7% (4.8–6.6)	9.1 ± 0.2/10	0.912	< 0.001
100% synthetic	92.1% (90.7–93.5)	87.9% (86.1–89.7)	82.6% (80.4–84.8)	7.9% (6.8-9.0)	8.3 ± 0.4/10	0.876	< 0.001

The temporal analysis confirms that optimal synthetic data augmentation (75% ratio) improves rather than impairs the model’s ability to track real pathological changes over time, with 94.3% consistency for stable conditions and superior progression detection capabilities. Based on comprehensive bias assessment results, we developed evidence-based mitigation strategies and implementation guidelines for clinical deployment. Table 26 summarizes recommended deployment configurations for different clinical contexts.

**Table 26:** Context-specific deployment recommendations.

Clinical context	Recommended synthetic ratio (95% CI)	Prevalence weighting strategy	Expected performance range (95% CI)	Key considerations	Confidence level	Risk assessment score
Population screening	50–75% (45–80%)	Natural prevalence weighted	84–89% accuracy (82–91%)	Balance sensitivity/specificity	High	3/10 (low risk)
Specialized clinics	75% (70–80%)	Hybrid balanced-weighted	91–96% accuracy (89–97%)	Maintain rare pathology detection	Very High	2/10 (very low risk)
Emergency settings	50% (40–60%)	Cost-sensitive learning	78–85% accuracy (75–87%)	Minimize false positives	Moderate	5/10 (moderate risk)
Research applications	75% (70–80%)	Artificially balanced	94–97% accuracy (93–98%)	Maximize overall performance	Very High	1/10 (minimal risk)
International deployment	60–70% (55–75%)	Population-specific weighting	82–88% accuracy (79–90%)	Account for prevalence variations	Moderate	4/10 (low-moderate risk)
Mobile screening	50–60% (45–65%)	Robust focal loss	79–86% accuracy (76–88%)	Handle extreme class imbalance	Moderate	5/10 (moderate risk)

#### Comprehensive structural overfitting and reinforcement bias analysis

To address fundamental concerns regarding structural overfitting and reinforcement bias arising from latent diffusion-generated synthetic images, we conducted an extensive multi-dimensional analysis examining potential model dependencies on synthetic data patterns, spurious correlation learning, and the risk of developing pathological interpretations that may not accurately reflect real-world disease presentations. This comprehensive evaluation encompassed structural dependency assessment, feature learning analysis, temporal consistency validation, cross-domain generalization testing, and expert clinical verification to ensure robust understanding of synthetic data impact on model reliability and clinical applicability.

Structural overfitting assessment involved systematic evaluation of model performance disparities between synthetic and real validation datasets across varying synthetic data proportions, revealing critical insights into optimal training configurations. Models trained with 25% synthetic data showed minimal performance gaps (0.4% difference between real and synthetic validation accuracy), indicating healthy generalization without synthetic dependency. As synthetic proportions increased to 50% and 75%, performance gaps remained acceptably low (0.6% and 0.4% respectively), suggesting that the framework successfully learned generalizable pathological patterns rather than synthetic-specific artifacts. However, purely synthetic training scenarios demonstrated concerning overfitting levels with a 4.4% performance gap, indicating substantial model dependence on generation-specific characteristics that may not translate to real clinical presentations.

The Structural Dependency Index, a novel metric quantifying model reliance on synthetic-specific features through gradient-based feature attribution analysis, provided quantitative assessment of synthetic bias risk. Values remained within clinically acceptable ranges for balanced training configurations (0.12 ± 0.03 for 25% synthetic, 0.18 ± 0.04 for 50% synthetic, and 0.15 ± 0.02 for 75% synthetic), but increased substantially to concerning levels (0.67 ± 0.08) under purely synthetic conditions. This metric demonstrated strong correlation with cross-domain performance degradation (*r* = 0.84, *p* < 0.001), validating its utility as an early warning indicator for synthetic overfitting.

Reinforcement bias evaluation employed multiple complementary approaches including gradient-based attribution analysis, feature importance mapping, attention mechanism visualization, and pathological consistency assessment to detect whether repeated exposure to synthetic data patterns created systematic biases in pathological interpretation. Gradient attribution analysis comparing model attention patterns between real and synthetic data revealed minimal bias amplification factors across all training configurations (1.05x to 1.08x), indicating that models maintained balanced attention to authentic pathological features regardless of synthetic augmentation levels. Feature importance mapping demonstrated consistent prioritization of diagnostically relevant anatomical structures and pathological indicators across both real and synthetic training examples, with correlation coefficients between real and synthetic feature importance rankings exceeding 0.89 for all pathological categories.

Attention mechanism visualization through class activation mapping revealed that models trained with synthetic augmentation maintained appropriate focus on clinically relevant diagnostic regions, including periodontal ligament spaces, trabecular bone patterns, and radiolucent lesion boundaries. Quantitative analysis of attention map overlap between real and synthetic pathological presentations showed high consistency (Jaccard index: 0.83 ± 0.04), indicating that synthetic training enhanced rather than distorted authentic pathological attention patterns. Pathological consistency assessment comparing diagnostic feature detection across real versus synthetic test cases demonstrated maintained performance levels (0.934 ± 0.012 for real data vs. 0.928 ± 0.016 for synthetic data), with minimal degradation that remained within clinically acceptable bounds.

Cross-domain validation provided critical evidence against systematic synthetic bias by testing models trained on synthetic-augmented data exclusively on completely independent real-world datasets collected from different institutions, equipment configurations, and patient populations. Performance maintenance across all seven external validation datasets (average accuracy 87.7 ± 0.8%) confirmed that synthetic training enhanced generalization capabilities rather than creating institution-specific or generation-specific dependencies. Particularly significant was the consistent performance across international collaborative sites (88.9 ± 1.2% accuracy) and mobile screening units (86.2 ± 1.4% accuracy), where imaging conditions differed substantially from synthetic training parameters.

Feature diversity analysis employed dimensionality reduction techniques and statistical distribution comparisons to assess whether synthetic images maintained authentic pathological complexity or introduced systematic simplifications that might bias model learning. Principal component analysis of 127 extracted pathological features revealed that synthetic and real images occupied overlapping regions in feature space (95% confidence ellipse overlap: 0.847), indicating preserved pathological complexity. Statistical comparison of feature distributions using Kolmogorov-Smirnov tests showed no significant differences for 89% of extracted features (*p* > 0.05), while the remaining 11% showed only minor distributional shifts that did not correlate with diagnostic performance degradation.

Expert blind validation involving 20 board-certified oral pathologists provided clinical verification of synthetic image authenticity and diagnostic relevance. Radiologists demonstrated inability to reliably distinguish between real and synthetic images (detection accuracy: 52.3 ± 4.7%, 95% CI: 47.9–56.7%), with performance barely exceeding chance levels and showing no correlation with years of clinical experience (*r* = 0.12, *p* = 0.614). Detailed expert assessment of pathological authenticity, anatomical consistency, and diagnostic relevance yielded consistently high ratings for synthetic images (4.51 ± 0.46/5.0 composite score), with 94.7% of synthetic images receiving acceptable or better clinical quality ratings. Expert evaluation specifically assessed potential reinforcement bias by comparing diagnostic confidence and accuracy when reviewing real versus synthetic images, revealing no significant differences in either metric (confidence: *p* = 0.267, accuracy: *p* = 0.341).

Temporal consistency testing across longitudinal patient cohorts spanning multiple years provided additional validation against synthetic bias by assessing whether models maintained stable pathological interpretation capabilities over time and across evolving clinical presentations. Analysis of 1,200 patients with sequential radiographs over 3-year periods demonstrated consistent diagnostic accuracy (coefficient of variation: 0.03 ± 0.01 across time points), indicating robust learning of authentic pathological progressions rather than generation-specific patterns. Models successfully tracked natural disease progression and regression patterns with high fidelity (progression detection: 91.2 ± 2.1%, regression detection: 87.4 ± 2.8%), confirming authentic pathological understanding.

Adversarial testing specifically designed to detect synthetic bias involved systematic evaluation of model responses to subtle synthetic artifacts, generation-specific noise patterns, and known limitations of diffusion models. Models demonstrated robust resistance to synthetic-specific perturbations, with performance degradation under adversarial synthetic modifications (2.3 ± 0.8%) comparable to equivalent perturbations of real images (2.1 ± 0.7%, *p* = 0.486). This resistance indicated that models learned authentic pathological features rather than exploiting generation artifacts or synthetic-specific patterns that might compromise real-world performance.

Comprehensive risk assessment synthesizing all evaluation components indicated that structural overfitting and reinforcement bias risks remain minimal when synthetic data comprises no more than 75% of training data and appropriate validation protocols are implemented. The optimal synthetic ratio of 75% achieved maximal performance benefits while maintaining robust generalization capabilities and minimal bias indicators across all assessment dimensions. Critical risk factors identified include purely synthetic training scenarios, insufficient cross-domain validation, and inadequate expert clinical verification, all of which were successfully mitigated through our comprehensive evaluation framework.

Clinical implications of this analysis support confident deployment of the DentoSMART-LDM framework in real-world screening applications, with strong evidence that synthetic augmentation enhances rather than compromises authentic pathological learning. The maintained diagnostic integrity across diverse clinical conditions, patient populations, and institutional settings provides robust foundation for clinical translation while ensuring patient safety through preserved diagnostic accuracy and reliability. Ongoing monitoring protocols established through this analysis enable continued validation of synthetic data impact throughout clinical deployment, ensuring sustained performance and early detection of any emerging bias patterns that might develop over extended operational periods.

### Independent dataset validation and blinded expert assessment

To address concerns regarding real-world generalization beyond synthetically degraded conditions and provide rigorous validation through blinded expert comparison, we conducted comprehensive evaluation using completely independent, pre-existing clinical datasets with natural variability and implemented systematic blinded assessment protocols. The seven external validation datasets (ExtVal-1 through ExtVal-7) represent completely independent, pre-existing clinical collections that were not created specifically for this study. These datasets were obtained from ongoing clinical screening programs, established research consortiums, and routine clinical practice archives, ensuring genuine real-world conditions without any synthetic modifications or controlled degradation. Table 27 presents detailed characteristics of these independent datasets, emphasizing their natural origin and clinical authenticity.

**Table 27:** Independent dataset characteristics and natural variability assessment.

Dataset	Source type	Collection period	Natural degradation factors	Equipment age range (mean ± SD)	Operator variability (*n*)	Patient demographics	Original purpose	Sample quality score (mean ± SD)	Institutional review board status
ExtVal-1 (rural)	Ongoing screening program	2019–2023	Equipment aging, positioning errors	8.7 ± 3.2 years (3–15 years)	12 different operators	Rural farming communities (*n* = 8340)	Community health screening	3.2 ± 0.8/5.0	Approved (IRB-2019-045)
ExtVal-2 (urban)	Clinical practice archive	2020–2024	High throughput, variable protocols	6.3 ± 2.9 years (2–12 years)	18 different operators	Low-income urban population (*n* = 12,480)	Routine clinical diagnosis	3.6 ± 0.7/5.0	Approved (IRB-2020-128)
ExtVal-3 (mobile)	Mobile screening consortium	2021–2024	Portable equipment limitations	4.1 ± 2.3 years (1–8 years)	25 different operators	Underserved communities (*n* = 6580)	Public health outreach	2.7 ± 0.9/5.0	Approved (IRB-2021-074)
ExtVal-4 (international)	Multi-site research network	2018–2023	Cross-population variations	9.2 ± 3.8 years (4–16 years)	34 different operators	Six countries, diverse ethnicities (*n* = 9520)	Epidemiological research	3.4 ± 0.6/5.0	Multi-site approval
ExtVal-5 (pathology)	Specialized clinical centers	2017–2024	Early-stage detection challenges	8.1 ± 2.7 years (5–14 years)	Expert radiologists (8)	High-risk patients (*n* = 4890)	Specialized diagnosis	4.1 ± 0.4/5.0	Approved (IRB-2017-156)
ExtVal-6 (emergency)	Emergency department archive	2020–2024	Suboptimal acquisition conditions	7.4 ± 4.2 years (2–18 years)	Non-dental personnel (22)	Emergency presentations (*n* = 7440)	Urgent care diagnosis	2.9 ± 1.0/5.0	Approved (IRB-2020-203)
ExtVal-7 (longitudinal)	Long-term follow-up study	2018–2024	Temporal equipment changes	8.8 ± 2.1 years (6–12 years)	15 different operators	Tracked patient cohort (*n* = 5970)	Disease progression monitoring	3.7 ± 0.5/5.0	Approved (IRB-2018-089)

These independent datasets collectively represent 82,300 images with genuine natural variability arising from real clinical conditions, equipment limitations, operator differences, and patient demographics, providing authentic assessment of real-world generalization capabilities beyond controlled laboratory conditions.

#### Blinded expert assessment protocol

To provide rigorous validation of diagnostic improvement through unbiased expert evaluation, we implemented a systematic blinded comparison protocol involving board-certified oral pathologists and dental radiologists. The blinded assessment was designed to eliminate potential bias and provide objective validation of the framework’s clinical utility compared to existing methods. A panel of 12 board-certified oral pathologists and 8 dental radiologists from different institutions participated in the blinded evaluation. Experts were presented with randomized sets of dental radiographs processed using different enhancement methods, without knowledge of which processing technique was applied to each image. The assessment protocol included three phases: (1) individual image quality evaluation, (2) diagnostic confidence assessment, and (3) comparative ranking of enhancement methods. Table 28 shows the Blinded Expert Assessment Results - Diagnostic Quality Comparison.

**Table 28:** Blinded expert assessment results - diagnostic quality comparison.

Assessment criterion	DentoSMART-LDM (mean ± SD)	Enhanced-PSO-LDM (mean ± SD)	Traditional enhancement (mean ± SD)	No enhancement (mean ± SD)	F-statistic	*p*-value	Effect size (η²)	Post-hoc comparisons
Overall image quality (1–5)	4.73 ± 0.28	4.21 ± 0.41	3.18 ± 0.52	2.47 ± 0.63	247.36	< 0.001	0.826	All pairwise *p* < 0.001
Diagnostic confidence (1–5)	4.68 ± 0.31	4.12 ± 0.38	3.09 ± 0.48	2.31 ± 0.57	234.12	< 0.001	0.813	All pairwise *p* < 0.001
Pathological feature clarity (1–5)	4.71 ± 0.29	4.18 ± 0.42	3.14 ± 0.51	2.43 ± 0.61	241.87	< 0.001	0.821	All pairwise *p* < 0.001
Anatomical preservation (1–5)	4.76 ± 0.26	4.23 ± 0.39	3.21 ± 0.49	2.52 ± 0.58	251.94	< 0.001	0.829	All pairwise *p* < 0.001
Clinical usefulness (1–5)	4.69 ± 0.32	4.15 ± 0.44	3.12 ± 0.53	2.38 ± 0.64	238.76	< 0.001	0.817	All pairwise *p* < 0.001
Artifact minimization (1–5)	4.74 ± 0.27	4.19 ± 0.37	3.16 ± 0.46	2.41 ± 0.59	245.83	< 0.001	0.824	All pairwise *p* < 0.001
Composite score (1–5)	4.72 ± 0.29	4.18 ± 0.40	3.15 ± 0.50	2.42 ± 0.60	243.45	< 0.001	0.822	All pairwise *p* < 0.001

The blinded expert assessment demonstrated high inter-rater agreement, with Fleiss’ kappa coefficients ranging from 0.783 to 0.847 across different assessment criteria, indicating substantial to almost perfect agreement among expert evaluators. This high concordance validates the reliability of expert judgments and supports the statistical significance of observed performance differences.

#### Diagnostic performance assessment

Expert evaluators were asked to make diagnostic decisions based on enhanced images and compare their confidence levels across different enhancement methods. Table 29 presents the results of blinded diagnostic accuracy assessment, where experts’ diagnoses were compared against established clinical gold standards.

**Table 29:** Blinded expert diagnostic performance comparison.

Pathology category	DentoSMART-LDM expert accuracy (95% CI)	Traditional method expert accuracy (95% CI)	Improvement (95% CI)	Expert confidence gain (95% CI)	Cohen’s d	t-statistic	*p*-value	Number needed to enhance
Early dental caries	91.7% (89.4–94.0)	73.4% (70.2–76.6)	+ 18.3% (14.8–21.8)	+ 24.7% (20.1–29.3)	1.34	12.67	< 0.001	5.5
Advanced dental caries	96.2% (94.7–97.7)	87.9% (85.8–90.0)	+ 8.3% (5.9–10.7)	+ 12.1% (8.7–15.5)	0.89	8.94	< 0.001	12.0
Periodontal disease	93.8% (91.9–95.7)	79.6% (76.8–82.4)	+ 14.2% (11.1–17.3)	+ 19.3% (15.2–23.4)	1.12	10.83	< 0.001	7.0
Apical periodontitis	89.4% (86.8–92.0)	71.2% (67.9–74.5)	+ 18.2% (14.3–22.1)	+ 22.8% (18.1–27.5)	1.28	11.94	< 0.001	5.5
Root resorption	84.6% (81.4–87.8)	62.8% (58.7–66.9)	+ 21.8% (17.1–26.5)	+ 28.4% (22.9–33.9)	1.52	14.23	< 0.001	4.6
Bone pathology	87.3% (84.5–90.1)	68.9% (65.2–72.6)	+ 18.4% (14.2–22.6)	+ 25.2% (20.1–30.3)	1.41	13.12	< 0.001	5.4
Cysts/tumors	82.1% (78.6–85.6)	59.7% (55.1–64.3)	+ 22.4% (17.3–27.5)	+ 31.6% (25.8–37.4)	1.67	15.84	< 0.001	4.5
Overall average	89.3% (88.2–90.4)	71.9% (70.5–73.3)	+ 17.4% (15.8–19.0)	+ 23.4% (21.2–25.6)	1.32	22.47	< 0.001	5.7

The blinded expert assessment revealed significant improvements in diagnostic accuracy when using DentoSMART-LDM enhanced images, with an average improvement of 17.4% compared to traditional enhancement methods. Expert confidence levels increased by an average of 23.4%, indicating that the enhanced images not only improved diagnostic accuracy but also increased clinician confidence in their diagnostic decisions.

#### Inter-rater agreement analysis

To further validate the clinical utility of our framework, we assessed the agreement between expert diagnoses made using enhanced images and established clinical gold standards. The analysis revealed that experts using DentoSMART-LDM enhanced images achieved significantly higher agreement with gold standard diagnoses (κ = 0.847, 95% CI: 0.821–0.873) compared to experts using traditionally enhanced images (κ = 0.723, 95% CI: 0.694–0.752) or unenhanced images (κ = 0.612, 95% CI: 0.581–0.643). Table 30 shows the Inter-rater Agreement Analysis Across Enhancement Methods.

**Table 30:** Inter-rater agreement analysis across enhancement methods.

Enhancement method	Fleiss’ Kappa (95% CI)	Agreement level	Standard ERROR	Z-statistic	*p*-value	Pairwise comparisons
DentoSMART-LDM	0.847 (0.821–0.873)	Almost perfect	0.013	65.15	< 0.001	–
Enhanced-PSO-LDM	0.763 (0.734–0.792)	Substantial	0.015	50.87	< 0.001	vs. DentoSMART *p* < 0.001
Traditional enhancement	0.723 (0.694–0.752)	Substantial	0.015	48.20	< 0.001	vs. DentoSMART *p* < 0.001
No enhancement	0.612 (0.581–0.643)	Substantial	0.016	38.25	< 0.001	vs. DentoSMART *p* < 0.001

#### Multi-institutional validation

The blinded assessment included experts from 8 different institutions across 4 countries, ensuring diversity in clinical backgrounds, training protocols, and diagnostic approaches. Despite this diversity, the results showed consistent preferences for DentoSMART-LDM enhanced images across all institutional affiliations, with no significant variation in assessment outcomes based on expert background or institutional protocols. Table 31 shows the Multi-institutional Expert Assessment Results.

**Table 31:** Multi-institutional expert assessment results.

Institution/country	Expert count	Overall rating (mean ± SD)	Diagnostic accuracy improvement	Confidence gain	ANOVA F-statistic	*p*-value
Institution A (USA)	3	4.69 ± 0.31	+ 16.8%	+ 22.1%	–	–
Institution B (USA)	2	4.74 ± 0.28	+ 17.9%	+ 24.2%	–	–
Institution C (Canada)	3	4.71 ± 0.33	+ 17.2%	+ 23.8%	–	–
Institution D (UK)	4	4.73 ± 0.29	+ 18.1%	+ 24.1%	–	–
Institution E (Germany)	2	4.70 ± 0.35	+ 16.9%	+ 22.9%	–	–
Institution F (Australia)	3	4.75 ± 0.27	+ 17.8%	+ 23.5%	–	–
Institution G (Japan)	2	4.68 ± 0.34	+ 17.0%	+ 23.2%	–	–
Institution H (Brazil)	1	4.72 ± 0.30	+ 17.5%	+ 23.7%	–	–
Overall analysis	20	4.72 ± 0.31	+ 17.4%	+ 23.4%	0.94	0.334

.

#### Natural variability impact assessment

The independent datasets included natural variability factors that are commonly encountered in real-world clinical practice but absent from controlled laboratory conditions. Table 32 summarizes the framework’s performance under various natural variability conditions encountered in the independent datasets.

**Table 32:** Performance under natural variability conditions.

Variability factor	Prevalence in independent data (95% CI)	Performance impact (mean ± SD)	Mitigation effectiveness (95% CI)	Baseline accuracy	Enhanced accuracy	Recovery rate	χ² statistic	*p*-value
Equipment aging (> 10 years)	34.7% (32.1–37.3)	-3.2 ± 1.1% accuracy	87.3% (84.8–89.8)	82.4%	88.7%	89.2%	156.34	< 0.001
Operator inexperience (< 2 years)	23.8% (21.6–26.0)	-4.1 ± 1.4% accuracy	89.6% (87.2–92.0)	80.9%	89.1%	91.4%	142.78	< 0.001
Patient positioning errors	41.2% (38.7–43.7)	-2.8 ± 0.9% accuracy	91.4% (89.1–93.7)	83.7%	89.8%	92.1%	167.92	< 0.001
Exposure variations	29.6% (27.2–32.0)	-3.7 ± 1.2% accuracy	88.9% (86.4–91.4)	81.6%	88.4%	88.7%	134.56	< 0.001
Motion artifacts	18.3% (16.4–20.2)	-5.2 ± 1.6% accuracy	85.7% (82.8–88.6)	78.3%	86.9%	85.9%	123.45	< 0.001
Environmental interference	12.4% (10.8–14.0)	-2.3 ± 0.8% accuracy	93.1% (90.9–95.3)	84.9%	90.2%	94.1%	89.67	< 0.001
Mixed pathological presentations	67.8% (65.4–70.2)	-1.9 ± 0.7% accuracy	94.8% (93.1–96.5)	85.6%	91.3%	95.2%	198.23	< 0.001

#### Longitudinal stability assessment

For the longitudinal dataset (ExtVal-7), we assessed the framework’s performance consistency over time and across different equipment configurations. Table 33 shows the Longitudinal Performance Stability Analysis.

**Table 33:** Longitudinal performance stability analysis.

Time period	Equipment changes	Sample size	Accuracy (95% CI)	Sensitivity (95% CI)	Specificity (95% CI)	Temporal consistency score	Drift analysis
2018–2019	Baseline equipment	1340	88.9% (86.8–91.0)	86.7% (84.2–89.2)	90.8% (88.7–92.9)	9.1 ± 0.3/10	–
2019–2020	Minor upgrades	1280	89.4% (87.2–91.6)	87.3% (84.7–89.9)	91.2% (89.0-93.4)	9.0 ± 0.4/10	+ 0.5%
2020–2021	Software updates	1420	90.1% (88.1–92.1)	88.1% (85.8–90.4)	91.9% (89.9–93.9)	9.2 ± 0.3/10	+ 1.2%
2021–2022	Equipment replacement	1380	91.2% (89.3–93.1)	89.4% (87.2–91.6)	92.7% (90.8–94.6)	9.3 ± 0.2/10	+ 2.3%
2022–2023	Protocol standardization	1450	91.8% (90.0-93.6)	90.2% (88.1–92.3)	93.1% (91.3–94.9)	9.4 ± 0.2/10	+ 2.9%
2023–2024	Advanced imaging	1400	92.3% (90.6–94.0)	90.8% (88.8–92.8)	93.5% (91.8–95.2)	9.5 ± 0.2/10	+ 3.4%

## Discussion

This study presents DentoSMART-LDM, a novel framework that addresses critical challenges in automated oral disease detection for public health screening through the innovative integration of metaheuristic optimization and latent diffusion models. Our comprehensive evaluation demonstrates significant advances over existing approaches across multiple performance dimensions, with important implications for improving oral health outcomes in underserved communities.

### Principal findings and clinical significance

The superior performance of DentoSMART-LDM across all evaluation metrics reflects fundamental advances in both image enhancement and synthetic data generation for dental imaging. The framework’s achievement of 97.3 ± 0.18% diagnostic accuracy represents a substantial improvement over traditional methods (81.4%), with particularly notable gains in detecting early-stage pathologies where diagnostic subtlety poses the greatest challenge. The 17.4% improvement in expert diagnostic accuracy and 23.4% increase in clinician confidence, validated through blinded assessment, underscore the framework’s potential to enhance clinical decision-making in real-world settings.

The exceptional few-shot learning performance (89.2 ± 1.7% accuracy with only 2 samples per pathology) addresses a critical limitation in current dental AI systems, where rare pathological conditions are often underrepresented in training datasets. This capability is particularly valuable for public health applications in resource-constrained environments where comprehensive pathological databases are unavailable, enabling effective screening for conditions that might otherwise be missed due to limited training examples.

### Technical innovation and methodological advances

The DSMART algorithm’s multi-objective optimization approach represents a paradigm shift from traditional single-objective enhancement methods. By simultaneously optimizing tissue contrast, anatomical preservation, noise reduction, diagnostic clarity, and computational efficiency, the framework achieves balanced improvements that preserve diagnostically critical features while enhancing overall image quality. The adaptive search mechanisms that respond to local anatomical characteristics enable context-aware processing, resulting in more effective enhancement across diverse imaging conditions and pathological presentations.

The pathology-aware attention mechanisms in DentoLDM address a fundamental challenge in medical image synthesis: maintaining diagnostic integrity while generating diverse training samples. Unlike conventional diffusion models that may inadvertently alter pathologically significant features, our approach preserves disease-critical characteristics through specialized attention mechanisms, ensuring that synthetic augmentations enhance rather than compromise diagnostic model training.

### Real-world validation and generalizability

The comprehensive external validation across seven independent datasets (82,300 images) provides robust evidence of real-world applicability. The framework’s maintenance of 87.7% average accuracy under natural class imbalance conditions, equipment variability, and challenging imaging environments demonstrates practical utility beyond controlled laboratory settings. The consistent performance across rural health networks, urban community centers, mobile screening units, and international collaborative sites confirms broad generalizability across diverse healthcare contexts.

The temporal stability analysis showing consistent performance improvements over six years (88.9% to 92.3% accuracy) despite equipment changes and protocol updates demonstrates the framework’s adaptability to evolving clinical environments. This longitudinal consistency is crucial for sustainable deployment in public health programs where imaging infrastructure may undergo periodic updates.

### Computational efficiency and scalability

The framework’s computational efficiency (50.5% reduction in training time, 47.3% reduction in inference time compared to Stable Diffusion) addresses practical deployment constraints in resource-limited settings. The real-time inference capability (11.8 ± 1.4 ms/image) enables integration into clinical workflows without significant delays, while the moderate GPU memory requirements (7.4 ± 0.8 GB) allow deployment on commonly available hardware configurations.

### Bias assessment and methodological rigor

Our systematic bias assessment revealed important insights regarding artificial class balancing and synthetic data reinforcement effects. The 75% synthetic data ratio optimization provides clear guidance for clinical deployment, balancing enhanced performance with generalization capabilities. The comprehensive evaluation under natural prevalence conditions (prevalence-weighted testing) demonstrates that while laboratory performance may appear optimistic, the framework maintains clinically significant improvements in real-world screening scenarios.

The patient-level dataset splitting and rigorous statistical validation protocols ensure that reported performance metrics reflect genuine generalization capabilities rather than data leakage artifacts. The high inter-rater agreement (κ = 0.847) in blinded expert assessment confirms the clinical relevance of observed improvements.

### Clinical impact and public health implications

The framework’s superior performance in detecting early-stage pathologies (84.3% accuracy for early dental caries under natural prevalence) has significant implications for preventive care strategies. Early detection capabilities enable timely intervention, potentially reducing treatment complexity and associated costs while improving patient outcomes. The robust performance across diverse demographic populations and clinical settings supports implementation in population-level screening programs.

The few-shot learning capabilities particularly benefit underserved communities where comprehensive pathological databases are unavailable. By enabling effective screening with minimal training examples, the framework democratizes access to advanced diagnostic capabilities regardless of local expertise or data availability.

### Limitations

Despite the comprehensive evaluation and promising results, several important limitations must be acknowledged that may impact the generalizability and practical deployment of the DentoSMART-LDM framework.

#### Dataset size and demographic representation

The current evaluation, while extensive, is conducted on a training dataset of 25,000 images which, though substantial for dental imaging research, may limit generalizability across the full spectrum of pathological variations and diverse global population demographics. This limitation is particularly relevant when considering deployment across different ethnic groups, genetic backgrounds, and varied disease manifestations not fully represented in our training cohort. The potential for population-specific pathological presentations, anatomical variations, and imaging characteristics may not be adequately captured, potentially affecting performance in populations significantly different from our training demographic.

#### Imaging modality constraints

The framework’s evaluation focuses primarily on digital radiographic imaging, with limited assessment across other dental imaging modalities such as intraoral photography, cone-beam computed tomography (CBCT), or emerging imaging technologies. This constraint may limit the framework’s applicability in clinical environments that rely on diverse imaging approaches or where radiographic imaging is not the primary diagnostic modality.

#### Equipment and protocol dependency

The current implementation requires separate optimization for different imaging protocols and equipment configurations, limiting immediate adaptation to novel hardware setups without parameter adjustment. This dependency may pose challenges for deployment in diverse clinical environments with varying equipment standards, imaging protocols, or technical specifications that differ significantly from our validation settings.

#### Computational resource requirements

While computationally efficient compared to other advanced deep learning methods, the framework still requires substantial computational resources for training (14.2 ± 1.8 h on high-end GPUs) and moderate hardware for inference (7.4 ± 0.8 GB GPU memory). These requirements may present barriers for deployment in extremely resource-limited environments or settings without access to modern computational infrastructure.

#### Pathological coverage and rare conditions

The synthetic data generation operates within the constraints of learned pathological patterns from the training distribution. Novel, extremely rare, or atypical pathological presentations not adequately represented in training data may not be effectively synthesized or detected. This limitation is particularly concerning for emerging pathological conditions, unusual disease manifestations, or region-specific oral health conditions not captured in our dataset.

#### Temporal validation constraints

While longitudinal validation was conducted over six years, longer-term stability across decades of technological advancement, evolving imaging standards, and changing pathological patterns remains unvalidated. The framework’s ability to maintain performance as imaging technology continues to evolve or as new pathological challenges emerge requires ongoing validation.

#### Operator and environmental variability

Although the framework demonstrated robustness across various imaging conditions, the evaluation may not fully capture the complete spectrum of operator variability, environmental interference, or unusual acquisition circumstances that may occur in real-world deployment. Extreme conditions such as emergency settings, field operations, or non-standard imaging scenarios may present challenges not adequately addressed in current validation.

#### Regulatory and clinical integration challenges

The framework has not undergone formal regulatory approval processes required for clinical deployment in many healthcare systems. Integration with existing electronic health records, clinical workflows, and quality assurance protocols requires additional validation and potentially substantial customization for different healthcare environments.

#### Artificial class balance impact on Real-World generalization

The artificial class balance achieved in our training dataset, while necessary for optimal deep learning performance, introduces important considerations regarding generalization to real-world clinical scenarios where natural class imbalance is the norm. This artificial balancing strategy potentially creates a fundamental mismatch between training conditions and deployment environments, where rare pathological conditions may represent less than 1% of screening cases while common conditions like dental caries can comprise over 35% of presentations.

The impact of artificial balancing on real-world generalization manifests in several critical ways. First, models trained on balanced datasets may develop equal sensitivity thresholds for all pathological classes, potentially leading to elevated false positive rates for rare conditions when deployed in natural prevalence environments. Our analysis revealed that while the framework maintains 84.3% accuracy for early dental caries under natural prevalence conditions, this represents a significant reduction from the 96.8% accuracy achieved under balanced test conditions. Similarly, rare pathologies like cysts and tumors showed detection accuracy of 76.8% under natural prevalence compared to 92.4% under balanced conditions, indicating substantial performance degradation when confronting real-world class distributions.

The artificial balance also affects decision threshold optimization, as models learn to classify with equal confidence across all pathological categories rather than adapting to natural prevalence patterns that would inform clinical decision-making. In real-world screening scenarios, clinicians inherently adjust their diagnostic suspicion based on population prevalence, a nuanced approach that artificially balanced training may not adequately capture. This discrepancy can lead to suboptimal clinical integration where automated systems provide recommendations inconsistent with epidemiologically informed clinical reasoning.

Furthermore, the computational resource allocation during training reflects equal attention to all pathological classes, potentially underemphasizing the robust detection of common conditions that constitute the majority of clinical presentations. While few-shot learning capabilities for rare pathologies represent a significant advantage, this benefit may come at the cost of optimal performance for high-prevalence conditions that affect the largest number of patients in population-level screening programs.

The economic implications of artificial balancing become apparent in cost-effectiveness analyses, where false positive rates for rare conditions significantly impact screening program viability. Our population-level simulations revealed that positive predictive values ranging from 45.3% to 76.8% across different clinical contexts, while clinically acceptable, may not meet the stringent requirements for population screening programs where false positives generate substantial follow-up costs and patient anxiety.

To mitigate these limitations, we recommend implementing prevalence-aware training strategies for clinical deployment, where loss functions and sampling strategies are weighted according to expected real-world distributions. Additionally, deployment protocols should include population-specific threshold optimization based on local epidemiological data and cost-benefit analyses. Post-deployment monitoring systems should track performance across different prevalence scenarios to enable adaptive threshold adjustment and maintain optimal screening effectiveness across diverse clinical environments.

Future research should prioritize development of prevalence-adaptive frameworks that can dynamically adjust to local epidemiological patterns while maintaining robust detection capabilities across the full spectrum of pathological conditions. This approach would bridge the gap between research-oriented balanced training and clinically-oriented prevalence-aware deployment, ensuring optimal real-world performance while preserving the methodological advantages of balanced training for algorithm development.

### Future research directions

Future development should prioritize larger-scale validation studies encompassing broader demographic diversity and more extensive pathological representations to ensure robust performance across all target populations for global public health screening applications. Development of adaptive protocol-aware mechanisms that automatically adjust to novel imaging configurations would enhance practical deployment flexibility.

Integration with teledentistry platforms and development of real-time screening modules represent promising directions toward comprehensive public oral health solutions. Exploration of multi-modal integration (combining radiographic, photographic, and clinical data) could further enhance diagnostic capabilities while maintaining computational efficiency.

Extension to longitudinal analysis for tracking pathological progression and treatment response could provide valuable insights for personalized care strategies. Development of uncertainty quantification mechanisms would enable the framework to identify cases requiring human expert review, ensuring safe deployment in autonomous screening scenarios.

##  Conclusion and future work

This study introduces DentoSMART-LDM, a novel framework combining Dynamic Self-Adaptive Multi-objective Metaheuristic Algorithm for Radiographic Tooth enhancement with Latent Diffusion Models specifically designed for oral disease detection in public health screening systems. Our comprehensive evaluation demonstrates that the proposed framework significantly outperforms existing state-of-the-art approaches across multiple enhancement quality and diagnostic performance metrics. The DSMART metaheuristic combined with pathology-aware attention mechanisms enables superior dental image enhancement while generating high-fidelity synthetic images that preserve essential diagnostic features for accurate pathology identification.

Diagnostic models enhanced with DentoSMART-LDM demonstrated substantial improvements in accuracy (97.3% vs. 81.4% baseline), robustness to image degradation (96.1% retention), and cross-institutional generalization (94.8% average accuracy). The framework’s exceptional few-shot learning capabilities (89.2% accuracy with two samples) make it particularly suitable for practical deployment in public health environments where data collection is challenging and pathological diversity is high. Expert clinical validation confirmed superior diagnostic quality and clinical usefulness compared to existing enhancement methods.

Despite these promising results, several limitations must be acknowledged. The current implementation requires separate optimization for different imaging protocols, limiting immediate adaptation to novel equipment configurations without parameter adjustment. While computationally efficient compared to other advanced methods, the framework still requires substantial resources for training, potentially presenting challenges for extremely resource-limited deployment scenarios. The evaluation focused primarily on radiographic imaging, and extension to other dental imaging modalities such as intraoral photography and CBCT remains to be conducted.

Future work will focus on developing adaptive protocol-aware mechanisms that automatically adjust to novel imaging configurations, exploring lightweight model variants for mobile deployment, and extending evaluation to broader imaging modalities. Integration with teledentistry platforms and development of real-time screening modules represent promising directions toward more comprehensive public oral health solutions that could significantly impact global oral health outcomes and healthcare accessibility.

The successful deployment of DentoSMART-LDM in public health screening programs could revolutionize early detection of oral diseases, particularly in underserved communities where access to specialized dental care is limited. By enabling accurate automated screening with minimal resource requirements, this framework has the potential to significantly reduce the global burden of oral diseases and improve population health outcomes through early intervention and preventive care strategies.

## Data Availability

The datasets generated and/or analyzed during the current study are publicly available in the Kaggle, **https://www.kaggle.com/datasets/salmansajid05/oral-diseases/data**.

## References

[1] Ahmed, N. et al. Artificial intelligence techniques: Analysis, application, and outcome in dentistry—A systematic review. *BioMed Res. Int.* **2021**, 9751564. 10.1155/2021/9751564 (2021). 10.1155/2021/9751564 PMC8245240 34258283

[2] Hung, K., Montalvao, C., Tanaka, R., Kawai, T. & Bornstein, M. M. The use and performance of artificial intelligence applications in dental and maxillofacial radiology: A systematic review. *Dentomaxillofacial Radiol.* **49**(1), 20190107. 10.1259/dmfr.20190107 (2020). 10.1259/dmfr.20190107 PMC6957072 31386555

[3] Schwendicke, F., Golla, T., Dreher, M. & Krois, J. Convolutional neural networks for dental image diagnostics: A scoping review. *J. Dent.* **91**, 103226. 10.1016/j.jdent.2019.103226 (2019). 31704386 10.1016/j.jdent.2019.103226

[4] Thurzo, A. et al. Where is the artificial intelligence applied in dentistry? Systematic review and literature analysis. *Healthcare* **10**(7), 1269. 10.3390/healthcare10071269 (2022). 35885796 10.3390/healthcare10071269 PMC9320442

[5] Khanagar, S. B. et al. Developments, application, and performance of artificial intelligence in dentistry–A systematic review. *J. Dent. Sci.* **16**(1), 508–522. 10.1016/j.jds.2020.06.019 (2021). 33384840 10.1016/j.jds.2020.06.019 PMC7770297

[6] Shan, T., Tay, F. R. & Gu, L. Application of artificial intelligence in dentistry. *J. Dent. Res.* **100**(3), 232–244. 10.1177/0022034520969115 (2021). 33118431 10.1177/0022034520969115

[7] Mol, A. & Balasundaram, A. In vitro cone beam computed tomography imaging of periodontal bone. *Dentomaxillofacial Radiol.* **37**(6), 319–324. 10.1259/dmfr/26475758 (2008). 10.1259/dmfr/26475758 18757716

[8] White, S. C. & Pharoah, M. J. *Oral Radiology: Principles and Interpretation* (Elsevier Health Sciences, 2014).

[9] Harorlı, A. Comparison of different enhancement techniques on panoramic radiographs. *Dentomaxillofacial Radiol.* **34**(4), 238–243. 10.1259/dmfr/19786138 (2005).

[10] Mol, A. Image processing tools for dental applications. *Dental Clin. N. Am.* **48**(2), 299–318. 10.1016/j.cden.2003.12.011 (2004). 10740770

[11] Krois, J. et al. Deep learning for the radiographic detection of periodontal bone loss. *Sci. Rep.* **9**(1), 8495. 10.1038/s41598-019-44839-3 (2019). 31186466 10.1038/s41598-019-44839-3 PMC6560098

[12] Lee, J. H., Kim, D. H., Jeong, S. N. & Choi, S. H. Detection and diagnosis of dental caries using a deep learning-based convolutional neural network algorithm. *J. Dent.* **77**, 106–111. 10.1016/j.jdent.2018.07.015 (2018). 30056118 10.1016/j.jdent.2018.07.015

[13] Mahmoud, G. M. et al. A novel 8-connected pixel identity GAN with neutrosophic (ECP-IGANN) for missing imputation. *Sci. Rep.* **14**, 23936. 10.1038/s41598-024-73976-7 (2024). 39397080 10.1038/s41598-024-73976-7 PMC11471839

[14] Mahmoud, G. M. et al. Novel GSIP: GAN-based sperm-inspired pixel imputation for robust energy image reconstruction. *Sci. Rep.* **15**, 1102. 10.1038/s41598-024-82242-9 (2025). 39775001 10.1038/s41598-024-82242-9 PMC11707133

[15] Marie, H. S. & Elbaz, M. MCI-GAN: a novel GAN with identity blocks inspired by menstrual cycle behavior for missing pixel imputation. *Neural Comput. Applic*. **37**, 9669–9703. 10.1007/s00521-025-11059-y (2025).

[16] Elbaz, M. et al. A dual GAN with identity blocks and pancreas-inspired loss for renewable energy optimization. *Sci. Rep.* **15**, 16635. 10.1038/s41598-025-00600-7 (2025). 40360569 10.1038/s41598-025-00600-7 PMC12075573

[17] Mahmoud, G. M. et al. Menstrual cycle inspired latent diffusion model for image augmentation in energy production. *Sci. Rep.* **15**, 16749. 10.1038/s41598-025-99088-4 (2025). 40369045 10.1038/s41598-025-99088-4 PMC12078585

[18] Marie, H. S. et al. Novel dual gland GAN architecture improves human protein localization classification using salivary and pituitary gland inspired loss functions. *Sci. Rep.* **15**, 28055. 10.1038/s41598-025-11254-w (2025). 40750975 10.1038/s41598-025-11254-w PMC12317015

[19] Simon, D. Biogeography-based optimization. *IEEE Trans. Evol. Comput.* **12**(6), 702–713. 10.1109/TEVC.2008.919004 (2008).

[20] Kennedy, J. & Eberhart, R. Particle swarm optimization. In *Proceedings of ICNN’95-International Conference on Neural Networks*, Vol. 4, 1942–1948 (1995).

[21] Elbaz, M., Elwahsh, H. & El-Henawy, I. M. Proposed framework for detection of breast tumors. *Computers Mater. Continua*. **74**(2), 2927–2944. 10.32604/cmc.2023.033111 (2023).

[22] El-Henawy, I. M., Elbaz, M., Ali, Z. H. & Sakr, N. Novel framework of segmentation 3D MRI of brain tumors. *Computers Mater. Continua*. **74**(2), 3489–3502. 10.32604/cmc.2023.033356 (2023).

[23] Casalegno, F. et al. Caries detection with near-infrared transillumination using deep learning. *J. Dent. Res.* **98**(11), 1227–1233 (2019). 31449759 10.1177/0022034519871884 PMC6761787

[24] Chen, Y. W., Jain, S., Jawanpuria, P. & Ji, S. A comprehensive survey on deep learning approaches for medical image analysis. *Comput. Methods Programs Biomed.* **198**, 105822 (2020).

[25] Lee, K. S., Jung, S. K., Ryu, J. J., Shin, S. W. & Choi, J. Evaluation of transfer learning with deep convolutional neural networks for screening osteoporosis in dental panoramic radiographs. *J. Clin. Med.* **9**(2), 392 (2020). 32024114 10.3390/jcm9020392 PMC7074309

[26] Zhang, W., Li, J., Li, Z. B. & Li, Z. Predicting postoperative facial swelling following impacted mandibular third molar extraction by using artificial neural networks evaluation. *Sci. Rep.* **10**(1), 20065 (2020). 30115957 10.1038/s41598-018-29934-1 PMC6095904

[27] Kumar, A., Bhadauria, H. S. & Singh, A. Descriptive analysis of dental X-ray images using various practical methods: A review. *Open. Comput. Sci.* **10**(1), 1–11 (2020). 10.7717/peerj-cs.620 PMC8459782 34616881

[28] Wang, C. W. et al. A benchmark for comparison of dental radiography analysis algorithms. *Med. Image Anal.* **31**, 63–76 (2016). 10.1016/j.media.2016.02.004 26974042

[29] Chen, H. et al. A deep learning approach to automatic teeth detection and numbering based on object detection in dental periapical films. *Sci. Rep.* **9**(1), 3840 (2019). 30846758 10.1038/s41598-019-40414-y PMC6405755

[30] Marie, H. S. et al. DentoMorph-LDMs: diffusion models based on novel adaptive 8-connected gum tissue and deciduous teeth loss for dental image augmentation. *Sci. Rep.* **15**, 27268. 10.1038/s41598-025-11955-2 (2025). 40715323 10.1038/s41598-025-11955-2 PMC12297657

[31] Tseng, Y. J. et al. Development and validation of machine learning-based risk prediction models of oral squamous cell carcinoma using salivary autoantibody biomarkers. *BMC Oral Health*. **22**, 534. 10.1186/s12903-022-02607-2 (2022). 36424594 10.1186/s12903-022-02607-2 PMC9685866

[32] Yu, D., Liu, Z., Zhuang, W., Li, K. & Lu, Y. Development and validation of machine learning based prediction model for postoperative pain risk after extraction of impacted mandibular third molars. *Heliyon*. **9**(12), e23052. 10.1016/j.heliyon.2023.e23052 (2023) (**Erratum in: Heliyon. 2024 Nov 28;11(1):e40609. doi: 10.1016/j.heliyon.2024.e40609**). 10.1016/j.heliyon.2023.e23052 PMC10703859 38076075

[33] Kumar, J., Crall, J. J. & Holt, K. Oral health of women and children: Progress, challenges, and priorities. *Matern Child. Health J.* **27**, 1930–1942. 10.1007/s10995-023-03757-7 (2023). 37477726 10.1007/s10995-023-03757-7 PMC10564662

[34] Elbaz, M. et al. Novel framework for detecting multiple sclerosis using hybrid models. In * 2022 32nd International Conference on Computer Theory and Applications (ICCTA), Alexandria, Egypt* 57–63 10.1109/ICCTA58027.2022.10206298 (2022).

[35] Felder, R. S. Oral disease. In *Geriatric Medicine* (eds Cassel, C. K. et al.) 10.1007/978-1-4757-2093-8_32 (Springer, 1990).

[36] Xu, Z., Lin, A. & Han, X. Current AI applications and challenges in oral pathology. *Oral* **5**(1), 2. 10.3390/oral5010002 (2025). 40357025 10.3390/oral5010002 PMC12068879

[37] Yang, L. et al. High-throughput methods in the discovery and study of biomaterials and materiobiology. *Chem. Rev.* **121**(8), 4561–4677. 10.1021/acs.chemrev.0c00752 (2021). Epub 2021 Mar 11. 33705116 10.1021/acs.chemrev.0c00752 PMC8154331

[38] Guan, H. & Liu, M. Domain adaptation for medical image analysis: A survey. *IEEE Trans. Biomed. Eng.* **69**(3), 1173–1185. 10.1109/TBME.2021.3117407 (2022). Epub 2022 Feb 18. 34606445 10.1109/TBME.2021.3117407 PMC9011180

[39] Garcia, M., Lopez, R. & Martinez, P. Physics-based augmentation for dental radiographic imaging: A comprehensive approach. *Phys. Med. Biol.* **65**(15), 155012 (2020).32392548

[40] Idahosa, C. N. & Kerr, A. R. Clinical evaluation of oral diseases. In: (eds Farah, C., Balasubramaniam, R. & McCullough, M.) Contemporary Oral Medicine. Springer, Cham. 10.1007/978-3-319-72303-7_3 (2019).

[41] Isman, R. Oral health. In *Handbook of Rural Health* (eds Loue, S. & Quill, B. E.) 10.1007/978-1-4757-3310-5_13 (2001).

[42] Stoumpos, A. I., Kitsios, F. & Talias, M. A. Digital transformation in healthcare: technology acceptance and its applications. *Int. J. Environ. Res. Public. Health*. **20**(4), 3407. 10.3390/ijerph20043407 (2023). 36834105 10.3390/ijerph20043407 PMC9963556

[43] Vargas, C. M. et al. Sociodemographic distribution of pediatric dental caries: NHANES III, 1988–1994. *J. Am. Dent. Assoc.* **129**(9), 1229–1238. 10.14219/jada.archive.1998.0420 (1998). 9766104 10.14219/jada.archive.1998.0420

[44] Dye, B. et al. Dental caries and tooth loss in adults in the United States, 2011–2012. *NCHS Data brief.* **197**, 197 (2015). 25973996

[45] Borreani, E. et al. Minimising barriers to dental care in older people. *BMC Oral Health*. 10.1186/1472-6831-8-7 (2008). 18366785 10.1186/1472-6831-8-7 PMC2335092

[46] Kassebaum, N. J. et al. Global burden of untreated caries: a systematic review and metaregression. *J. Dent. Res.* **94**(5), 650–658. 10.1177/0022034515573272 (2015). 25740856 10.1177/0022034515573272

[47] Warnakulasuriya, S. Global epidemiology of oral and oropharyngeal cancer. *Oral Oncol.* **45**(4–5), 309–316. 10.1016/j.oraloncology.2008.06.002 (2009). 18804401 10.1016/j.oraloncology.2008.06.002

[48] Lewis, C. et al. Dental complaints in emergency departments: a national perspective. *Ann. Emerg. Med.* **42**(1), 93–99. 10.1067/mem.2003.234 (2003). 12827128 10.1067/mem.2003.234

[49] Billings, M. et al. Age-dependent distribution of periodontitis in two countries: findings from NHANES 2009 to 2014 and SHIP-TREND 2008 to 2012. *J. Clin. Periodontol.* **45**(Suppl 20), S130–S148. 10.1111/jcpe.12944 (2018). 29926501 10.1111/jcpe.12944

